# A Comprehensive Review of the Recent Developments in Wearable Sweat-Sensing Devices

**DOI:** 10.3390/s22197670

**Published:** 2022-10-10

**Authors:** Nur Fatin Adini Ibrahim, Norhayati Sabani, Shazlina Johari, Asrulnizam Abd Manaf, Asnida Abdul Wahab, Zulkarnay Zakaria, Anas Mohd Noor

**Affiliations:** 1 Faculty of Electronic Engineering & Technology, Universiti Malaysia Perlis, Arau 02600, Malaysia; 2 Center of Excellance Micro System Technology, Universiti Malaysia Perlis, Arau 02600, Malaysia; 3 Collaborative Microelectronic Design Excellence Centre, Universiti Sains Malaysia, Gelugor 11800, Malaysia; 4 Department of Biomedical Engineering and Health Sciences, Universiti Teknologi Malaysia, Johor Bahru 81310, Malaysia; 5 Sports Engineering Research Center, Universiti Malaysia Perlis, Arau 02600, Malaysia

**Keywords:** sweat-sensing applications, wearable device, real-time measurement, continuous and non-continuous flow

## Abstract

Sweat analysis offers non-invasive real-time on-body measurement for wearable sensors. However, there are still gaps in current developed sweat-sensing devices (SSDs) regarding the concerns of mixing fresh and old sweat and real-time measurement, which are the requirements to ensure accurate the measurement of wearable devices. This review paper discusses these limitations by aiding model designs, features, performance, and the device operation for exploring the SSDs used in different sweat collection tools, focusing on continuous and non-continuous flow sweat analysis. In addition, the paper also comprehensively presents various sweat biomarkers that have been explored by earlier works in order to broaden the use of non-invasive sweat samples in healthcare and related applications. This work also discusses the target analyte’s response mechanism for different sweat compositions, categories of sweat collection devices, and recent advances in SSDs regarding optimal design, functionality, and performance.

## 1. Introduction

**Figure 1:**
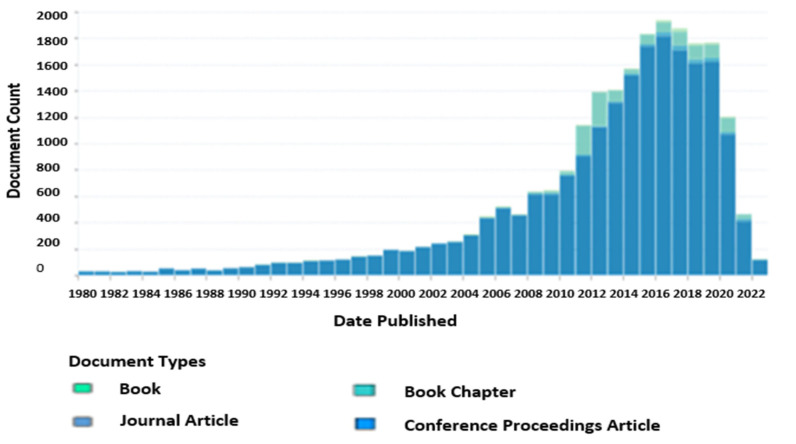
Scholarly work of sweat analysis from 1980 to 2021 based on lens.org [29].

Exploration of sweat compositions and analysis has gained great interest in the last few decades, as sweat contains extensive biochemical information that could indicate the body’s health state. It has led to the generation of extensive attention towards the development of sweat-sensing devices (SSDs) as a diagnostic biofluid. A growing number of research studies that have been reported on analyzing various sweat biomarkers for use in a broad range of clinical diagnostic purposes, including health concerns, nutritional imbalance, drug abuse, and many more [1,2,3,4], have seen a ten-fold rise in this field, as shown in Figure 1. However, there are still gaps in the structured content on related SSD applications based on the current existing review articles, such as insufficient information about a few sweat biomarkers, especially hormones, drugs, nicotine, and vitamin C, and inadequate detailed explanation of the respective application concepts related to these biomarkers and a lack of a fundamental response mechanism of chemical sensors that enable users to understand the process of the target analyte’s detection for various sweat compositions [5,6,7,8,9,10,11]. Despite these limitations, the existing reviews seemed to disregard addressing issues such as an analysis effect formation mixing new and old sweat samples, which can be a daunting challenge for acquiring a high precision in sweat analysis. On the other hand, the sampling method significantly impacts the accuracy and reliability of sweat analysis results [12]. Meanwhile, sweat sensors cannot differentiate between fresh and old sweat because it only measures the sample that comes into contact with its surface in real-time [13]. It may show incorrect dynamic changes in analyte concentration, resulting in an invalid sweat analysis. Utilizing microfluidic devices can solve this problem [14], but some of the designs are limited in processing volume capacity and face other challenges, for example, disposable design, low flow rates of sweat samples into the channel, difficulty in maintaining continuous sweat flow that can end up causing backflow, irritation for long measurements because sweat secretion on the skin can be blocked by its water-resistant materials, and complexity in integrating the sensor into its design while maintaining sensor stability [15,16]. There are very few recent review articles that have addressed this mixing of sweat in depth by specific SSD designs of continuous and non-continuous flow. Moreover, several review articles have commonly reported this issue by only pointing it out as a challenge of sweat-based continuous health monitoring platforms [12,17,18,19,20]. In addition, some articles have typically proposed the use of a microfluidic device to minimize the effects of blending and the continuation of sweat collection [21,22]. In contrast, this article provides a comprehensive review that addresses the gaps by discussing a critical overview of the analysis of mixed new and old sweat in SSDs, including the materials used, working principles, and the parameters measured for the proposed designs of sweat collection devices. Additionally, existing reviews have not sufficiently emphasized the recent advancements in SSD design, functionality, and performance for each primary component, including sweat collection devices, sensors, and electronic devices. The framework of many review articles specifically concentrates on reviewing recent technologies involved in electronic or novelty materials used in electrode sensors rather than an overview of possible progressive features for each main SSD component [23,24,25,26,27,28].

This paper comprehensively reviews wearable sweat-sensing devices’ recent development and limitations, focusing on continuous-sweat flow (CF) and non-continuous-sweat flow (NCF) analysis. The novel contribution of this paper is the mechanism CF and NCF of SSDs with various types of sweat collection devices. It is considered novel because this analysis has not yet been covered in previous review papers. There are five sections that are presented in non-invasive SSD studies that provide reliable opportunities for the continuous tracking of health status. Section 1 begins with an introduction that highlights the gaps in the studies of existing reviews on relevant important topics related to SSDs. Section 2 highlights sweat applications that influence biochemical sensor design for the five main types of sweat compositions (e.g., electrolytes, hormones, metabolites, micronutrients, and exogenous chemicals) that contain various biomarkers. A summary chart of sweat biomarkers with their potential applications and graphic mechanisms of sweat sensing are also illustrated in this section. Section 3 discusses the types of sweat collection devices and their principles, including devices with CF and NCF types. In addition, the underlying design mechanisms of CF and NCF are discussed in detail. Section 4 describes the recent advances in wearable SSD optimal design, functionalities, and performance. This section is divided into three main components based on the latest development of SSDs in clinical sweat research, along with summarizing tables. Lastly, Section 5 provides the conclusion and future research directions. In general, this review paper aims to understand the current structure of SSDs and provide well-organized theory mechanisms and previous works of sweat analysis for future applications.

## 2. Applications of Analytes in Sweat

**Figure 2:**
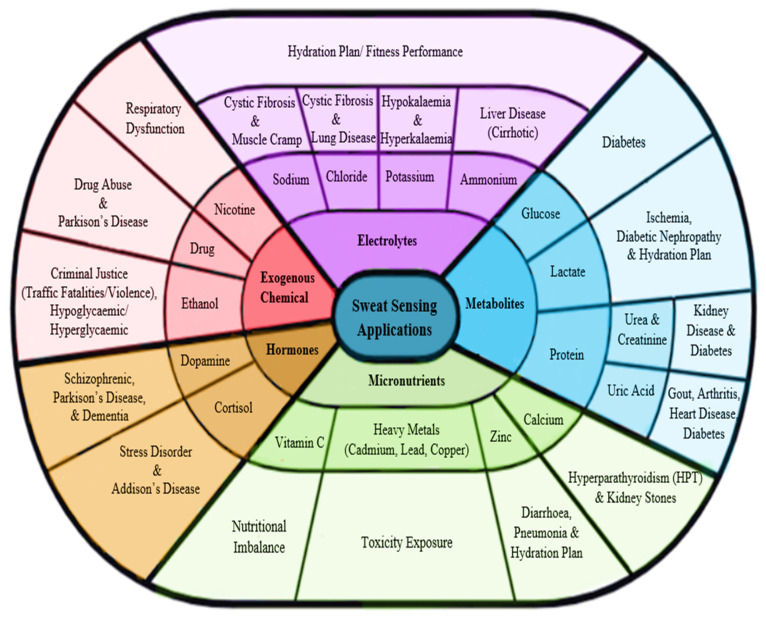
Analysis of sweat-sensing applications.

In this section, the potential sweat-sensing applications, including disease and health states related to sweat contents, are explored and investigated. Figure 2 represents the summary of the sweat applications by five primary types with their respective biomarkers based on the recent works and their significant role in clinical diagnostics. Sweat contents are highly personalized for each human being [30,31]. Therefore, each person comprises different physiological data of biomarkers that has advantages in various analyses of sweat, which has helpful information on human physiology, health, and performance. Interestingly, many biomarkers found in blood are also found in sweat, even at lower concentrations.

Electrolytes are often popular biomarkers that have been used in sweat applications, especially in sports performance and the diagnosis of some diseases. For instance, sodium [32,33,34], chloride [35], potassium [32], and ammonium [33,36] ions are the most frequent sweat ions detected by electrochemical sensors during physical exercise. It is important to monitor these ion measurements because they decrease over time during physical training. This analysis, such as the body hydration plan, allows athletes to effectively plan their training without overstressing their bodies, which causes injuries and reduces performance [34]. Therefore, hydration planning is essential to ensure athletes maintain their bodies with optimum stamina to exercise consistently during prolonged exercise. Other than that, abnormal electrolyte levels in sweat can also be an indicator of health problems, deterring health issues that could negatively impact overall well-being. For example, Hall et al. reported that children were considered positive for cystic fibrosis (CF) if their sweat contained one of the conditions of either sodium being greater than 60 mmol/L or chloride being greater than 70 mmol/L [37]. A high chloride level in sweat indicates CF disease, which is typically caused by abnormalities affecting certain glands that reabsorb chloride, resulting in severe lung devastation [38]. Additionally, a sodium deficit can cause heat cramps [39], and if this continues for an extended period of time, it may result in serious health problems such as hyponatremia [33]. Meanwhile, potassium in sweat can be used to determine the potassium level in blood plasma, whether an abnormal deficiency (hypokalemia) [40,41] or an excessively high level (hyperkalemia) [41]. Severe hypokalemia increases the risk of peripheral nerve disorders, falls, periodic paralysis, and chronic kidney disease [42]. Hyperkalemia impacts cardiac arrest, nausea, irritability, and decreased urine output [43]. For sweat ammonia applications, its levels in sweat can be used to trace liver diseases such as cirrhosis [44,45] because cirrhotic patients have elevated ammonia concentrations in their blood plasma and sweat compared to normal individuals.

Sweat biomarkers of metabolites have also been greatly studied for sweat analysis applications. There have been extensive efforts to develop non-invasive sweat-based glucose monitoring methods. Excessive glucose levels in sweat or blood, in particular, are associated with the most common chronic disease, diabetes [46]. The majority of researchers have typically implemented a conventional glucose sensor to trigger and measure the glucose concentration released from sweat using reverse iontophoresis (RI) [47,48,49]. In addition, glucose levels also can be detected in other biomolecules, including ascorbic acid, uric acid, and lactic acid [48,49]. In certain studies, they proved that drugs in human sweat such as acetaminophen, acetylsalicylic acid, and metformin can be alternatively used to sense glucose [50,51,52,53]. Bandodkar et al. utilized a tattoo-based epidermal diagnostic device with the RI technique for measuring sweat glucose non-invasively. They demonstrated that the device had good selectivity toward glucose in the presence of ascorbic acid, uric acid, and acetaminophen [48]. Apart from glucose, lactate is another biomarker that is relevant to potential clinical application for the analysis of metabolites in sweat. There are studies that strongly believe that sweat lactate is potentially reliable as a biomarker that can replace traditional blood lactate sampling by developing an electrochemical sensor that is highly sensitive to lactate concentration [33]. This opens up the possibility of the measurement of an individual’s general health status, such as pressure ischemia and poor oxidative metabolism [54]. In the meantime, Seki et al. developed an SSD for tracking sweat lactate to examine the relationship between the lactate threshold (LT) and ventilatory threshold (VT) [55]. They reported that the sweat LT was well correlated with the blood LT and the VT. The continuous monitoring of sweat lactate concentrations during exercise can provide additional information for quickly detecting the VT for use in related applications. For example, their device could possibly replace the current clinical practice of standard respiratory gas analysis by performing exercise tests on patients with heart disease. Although standard respiratory gas analysis is non-invasive, VT assessment using this method requires expert guidelines, and the analyzer device is expensive [56,57].

Sweat proteins are interest to many researchers for analyzing and investigating their biomarkers related to the early diagnosis and treatment of disease. Zhang et al. developed a microfluidic colorimetric SSD to analyze the sweat rate, urea, and creatinine concentration for indications relevant to kidney disease [58]. They added a pH indication value sensor to determine the different phases of chronic kidney disease. In addition, Sudha et al. developed an SSD to monitor sweat urea for the early detection of diabetic nephropathy [59]. Along with urea and creatinine, uric acid (UA) has also been proven to have a good relationship between blood and sweat concentrations [60]. Yang et al. developed a wearable sweat sensor that detects low levels of UA, tyrosine, and other vital sign parameters including temperature and respiration rate [61]. This device can be used to diagnose gout and metabolic disorders, which enables the analysis of the levels of uric acid in sweat that are higher in patients with gout than in healthy individuals and shows a similar result pattern using blood measurement. In addition to that, Wei et al. developed an SSD using an advanced fiber-structure sensing interface, offering an approach for the wide-ranging measurement of sweat uric acid [57]. They found a strong linear relationship between the biosensor’s current output and the uric acid concentration, demonstrating a highly precise and selective concentration of uric acid present in artificial sweat. Their SSD may be relevant to uric acid applications equivalent to abnormal UA levels in serum with a range of medical conditions. In a clinical test, abnormal uric acid in the blood can diagnose diseases such as type 2 diabetes, heart disease, kidney disease, arthritis, and gout.

Micronutrients (mineral ions and vitamins) are also promising in sweat analysis. Nyein et al. reported that the calcium concentration in sweat rises as pH falls due to the body’s lactic acid levels decreasing during cycling [62]. Calcium concentration is usually measured in urine and sweat for the diagnosis of kidney stones and hyperparathyroidism (HPT). Moreover, many heavy metals are micronutrients, such as chromium (Cr), copper (Cu), cobalt (Co), lead (Pb), selenium (Se), Vanadium (V), and zinc (Zn), which are necessary in very small quantities in humans [63]. Therefore, applications such as monitoring body toxicology from micronutrients for the early detection of heavy metal exposure by non-invasive assessment are essential. Gao et al. developed an SSD capable of tracking multiple heavy metals such as copper, zinc, mercury, cadmium, and lead concurrently and selectively through sweat and urine samples [64]. These heavy metals in the human body can indicate several diseases. For example, Wilson’s disease can result from excessive copper accumulation in the body [65]. On the other hand, lower copper levels can cause anemia and osteoporosis. Low zinc levels in the body are important for diagnosing diarrhea and pneumonia [66], while long-term exposure to high zinc levels can damage the liver and even the heart and lower the number of pancreatic enzymes. Additionally, mercury, lead, and cadmium are known toxic elements that can harm various organ systems in humans including the nervous, immune, and cardiovascular systems [67]. Therefore, determining how many heavy metals a person has been exposed to through non-invasive sweat can detect a variety of diseases or disorders much quicker than through blood analysis. Recently, wearable non-invasive biosensors for monitoring and analyzing precision nutrition such as vitamin C in sweat have been widely explored for avoiding nutritional imbalances and affording individualized advice for achieving and maintaining healthy nutrient concentrations [68,69,70].

Hormones such as cortisol and dopamine (DA) are also found in sweat and are frequently used to determine the responses of health status. Cortisol is important for diagnosing Addison’s disease, since patients with this condition have inadequate cortisol levels [71,72]. Elevated cortisol levels over prolonged periods have been linked to depression, hypertension, diabetes, and obesity [73]. Most studies have developed an SSD to monitor sweat cortisol levels for physiological assessment directly related to physical and mental stress during exercise and rest [73,74]. Meanwhile, dopamine (DA) is an important neurotransmitter in the central nervous system, cardiovascular system, and kidney, and any abnormality in DA levels is highly associated with many diseases and disorders including schizophrenia, Parkinson’s disease, and dementia [75,76]. Lei et al. constructed the first successful ultrasensitive dopamine biosensor based on manganese doping approach, providing a high selectivity of dopamine detection using a buffer solution of blood and artificial sweat [77]. The detection sensitivity of DA was later improved by Anshori et al. using the nanocomposite biosensing of functionalized multi-walled carbon nanotubes and silver nanoparticles [78]. Their biosensor demonstrated good DA selectivity and sensitivity, which is promising for the detection human DA in sweat and urine.

Sweat samples can reveal the past intake of exogenous chemical xenobiotics such as alcohol, drugs, and nicotine. In sweat alcohol applications, many researchers have developed wearable epidermal alcohol sensor systems that typically use iontophoresis to induce sweat by delivering the drug pilocarpine across the skin followed by a sensor for detecting sweat ethanol [79,80,81]. Sweat ethanol levels can provide information about alcoholism and represent unhealthy alcohol consumption leading to potential accident-involved vehicle crashes or violence. The analysis of sweat ethanol using an SSD is more reliable than a breath analyzer. The results of alcohol intake using a breath analyzer is highly susceptible to errors in alcohol vapor associated with consumer products (e.g., breath freshener, mouthwash, and e-cigarette smoking, as the e-liquid may contain ethanol) [82]. In addition, drinking alcohol affects blood sugar levels in patients and makes insulin less effective [83,84,85]. Bhide et al. demonstrated the sensor’s ability to continuously track alcohol and glucose levels in passive perspiration via iontophoresis [86]. Diabetic and pre-diabetic patients are able to manage their alcohol consumption by continuously assessing their glycemic content to circumvent adverse health effects.

Sweat testing for drugs could be used in sports competitions to detect illegal drugs taken by an athlete [87]. The sweat analysis method using sensor patches has been proposed as an alternative to urine-based drug tests because on-the-spot or real-time analysis and testing have become attractive for uncovering past drug abuse. Tai et al. introduced wearable sweat-sensing modules for the real-time tracking of methylxanthine drugs, specifically caffeine (a safe drug), to effectively provide and alert users of their insight into caffeine level consumption [3]. Caffeine is an ergogenic drug prohibited in sports events and tournaments, requiring participants to undertake a standard urine caffeine test before participating in competitions [88]. It has been reported that urine caffeine concentration correlates with plasma and sweat caffeine concentrations [89]. Lastly, sweat nicotine can be simply used for smoking cessation therapies, a straightforward measure of nicotine dosage released through smokers’ sweat using a wearable SSD. Mehmeti et al. reported that electrochemical nicotine sensors of the sweat could be utilized to discriminate accurately between heavy smokers and light smokers [90]. Tai et al. also designed a wearable sweat sensor for nicotine monitoring to quantitatively assess the wearers’ exposure to smoking between active and passive smokers (non-smoking individuals) [91]. They investigated the variation in chemical compositions and concentrations over time of commercial cigarettes to estimate the side effects on health, both for active smokers and those exposed to tobacco smoke inadvertently. Cigarette ingredients’ adverse physiological and psychological effects can result in sleep disorders, cardiovascular disease, addiction, diabetes, and respiratory dysfunction [92].

**Figure 3:**
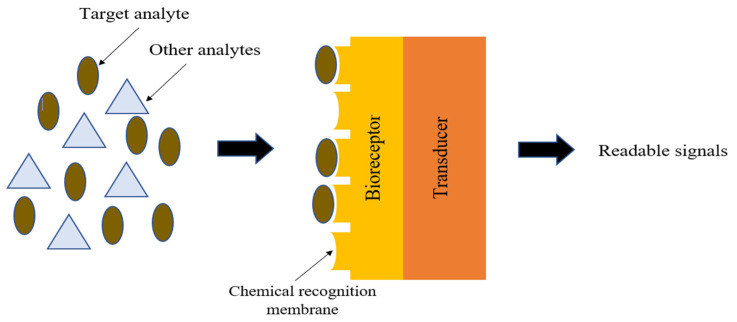
Fundamental response mechanism of sweat sensing.

Current sweat-sensing applications are based on the concept of a biochemical sensor, which is inspired by the elements in a fundamental response mechanism for sweat-sensing. A biochemical sensor is an analytical tool that transforms chemical or biochemical information, such as the concentration of sweat analytes, into an electrical signal (for an electrochemical sensor) or optical signal (for a colorimetric and fluorescence sensor). The elements that are involved in this mechanism are sweat biomarkers, a bioreceptor, and a transducer. There are two electrodes in a single electrochemical sensor, commonly forming ion-selective electrodes (ISEs): a working electrode and a reference electrode. Each electrode is drop-cast with their respective specific ion-selective membrane (ISM) solutions to form ISEs. This ISM solution can be prepared using different materials depending on the target biomarker (e.g., sodium, glucose, uric acid, levodopa, cortisol, or calcium) needing to be detected. This solution is composed of an ionophore and a lipid-soluble chemical species. The ionophore serves as a transducer by inducing ionic activity, which provides a specific electric signal [93]. Meanwhile, a bioreceptor is a lipid-soluble chemical species that can bind to and transport a particular ion across a membrane. For sweat sensing, its mechanism is closely related to the employed model of a lock and key. In this analogy, the lock is the bioreceptor, and the key is the target analyte. As shown in Figure 3, a bioreceptor’s chemical recognition membrane can identify a target analyte’s characteristics based on the sensitivity of its classification to a matching paired physical structure pattern. This bio-recognition binding system does not allow other analytes to cross the surface of the detection region. The transducer then converts the specific ion activity in a sample into a readable signal (electrical/optical). Measurement techniques such as potentiometry, voltammetry, electrical impedance spectroscopy (EIS), and capacitance are used to convert the output of an electrochemical sensor into a readable signal. For optical sensors, the direct analysis of color changes allows them to function with or without a transducer. Optical sensors incorporating transducers are typically supported by machine learning to extract useful data, as reported in a prior study [94].

## 3. Sweat-Sensing Device (SSD)

**Figure 4:**
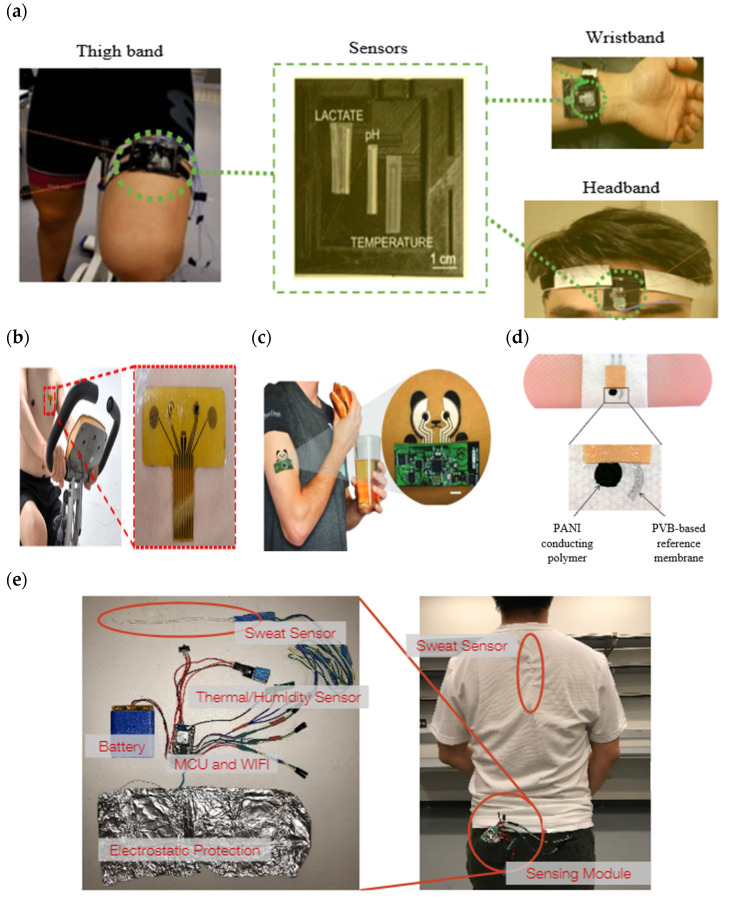
Types of wearable devices in sweat-sensing devices. (**a**) Sweatband [54]; (**b**) skin patch; reprinted from [104], copyright (2022), with permission from Elsevier. (**c**) Tattoo patch [106]; (**d**) bandage of epidermal patch [125] (Reused with the permission of copyright 2014, John Wiley, and Sons). (**e**) Textile [114].

A sweat-sensing device requires a wearable device for the temporary attachment of its main components, including sensors, sweat collection devices, and electronic devices to the body’s skin region. There are three primary types of wearable devices that are most commonly found in sweat applications: sweatbands [95], epidermal patches [96], and textiles [97]. Several factors to be considered in selecting which types of wearable devices are best suited for use in SSDs include the skin surface on a body location, sample collection techniques, and environmental conditions, whether on dry land or in aquatic exercise. For example, a sweatband is appropriate for wearing on specific body parts such as the wrist [98], arm [32], back [99], or forehead [100], where the bands can be tightened, as shown in Figure 4a. In addition, most of the literature prefers wearable sweatbands on the arm for cystic fibrosis tests during the conducting of conventional pilocarpine iontophoresis and sweat collection samples [101,102]. The band can be worn repeatedly with reusable electrodes during pilocarpine iontophoresis to trigger perspiration at any time due to the ease of detachment and reattachment of the electrodes from the band.

An epidermal patch is characterized by a disposable style of adhesive skin tape. It is low-cost, making it a practical SSD in a disposable format [103]. Wearable epidermal patches in SSDs typically come in various forms, such as skin patches [104], tattoos [105,106], and bandages [107,108], as shown in Figure 4b–d. The elements of substrate epidermal patches can promote strong skin adhesion, high mechanical strength, stretchability, and resilience in water conditions to manipulate physical skin performance. Moreover, an epidermal patch has a high flexibility for wearing on any part of the body’s skin and a good adaptability in high-intensity exercise. Furthermore, they are versatile enough to be worn for use in water sports such as swimming [109]. Reeder et al. proposed an epidermal patch for colorimetric sensing integrated with microfluidic and water-proof electronic systems that can perform real-time physiological measurements on swimmers and dryland athletes [110]. In particular, most colorimetry sensing applies epidermal patches as wearable devices, as this device can be used for the single-shot measurement of sweat biomarkers once they change color, as reported by previous studies [111,112].

A textile-based sensor has advantages over sweatbands and epidermal patches in terms of substrate washability when exposed to humidity and dirt. Moreover, the textile can serve either as a sweat collection device by absorption or as a wearable device. Recent textile-based sensing devices have a fitted sensor on regular cloth such as a t-shirt [113,114], as shown in Figure 4e. Thus, they offer the advantages of being comfortable and allowing users to wear in any regular clothes. Recently, some SSDs have been woven with sensor and electronic components into highly stretchable fibers, making them suitable for applications requiring high-motion applications. The detection of motion and physiological signals by a fitted sensor into a t-shirt also enable the easy examination of numerous physiological valuable data, including a person’s movement for the detection of Parkinson’s disease and stress levels [115,116,117]. Recently, several technologies and materials used in textile-based sensing devices have been improved to ensure they can operate under intense mechanical tension during regular activities and be reused without interfering with the analytical performance of a sensor after washing. Wicaksono et al. [113] and Martinez-Estrada et al. [118] developed a highly robust sensor-based textile that integrates with electronic component reusability after washing. A smart textile comprises water-resistant and detachable electronics for the convenience of washing, as well as a comfortable fabric sensor to prevent skin irritation for long-term monitoring [119].

An SSD can be composed of a single sensor or a combination of sensors with certain types of sweat collection devices to perform sweat analysis. A single sensor in an SSD is designed for direct skin contact to detect and measure sweat biomarkers such as metabolites that quickly degrade over time [120]. Thus, adopting a sweat collector, particularly a serpentine microfluidic device for analyzing proteins, is undesirable because sweat flow into a microchannel is time-consuming [121]. However, a sensor that has direct contact with the skin can irritate due to a rough sensor surface and contamination by perspiration from nearby areas. Combining a sensor with certain types of sweat collection devices can overcome these limitations. For example, utilizing paper-microfluidic integration with a sensor can prevent skin inflammation and contamination [122]. Moreover, the addition of paper to a microfluidic device is able to increase the flow rate of the transportation of sweat analytes into a sensor surface by absorption [123]. Therefore, sweat collection devices are essential components that can be added to an SSD. In addition, human sweat glands have small duct diameters, which are 5–40 μm for secretory coils and 10–20 μm for dermal ducts and upper coiled ducts [124], that limit the volume of sweat secretion. As a result, a sweat duct secretes a tiny portion of sweat from the bottom duct into the upper coiled duct region, with a microliter volume of total sweat being released at the skin’s surface. Hence, micro-collector devices are regularly required in biofluid testing to accumulate sample volumes in a microliter range or smaller for analysis, making them ideal for non-invasive diagnostic applications where the sample quantity is relatively small. Sweat collection devices have been recently utilized in the form of microfluidic devices, absorbent-based materials, and microneedle injection with transdermal delivery.

**Figure 5:**
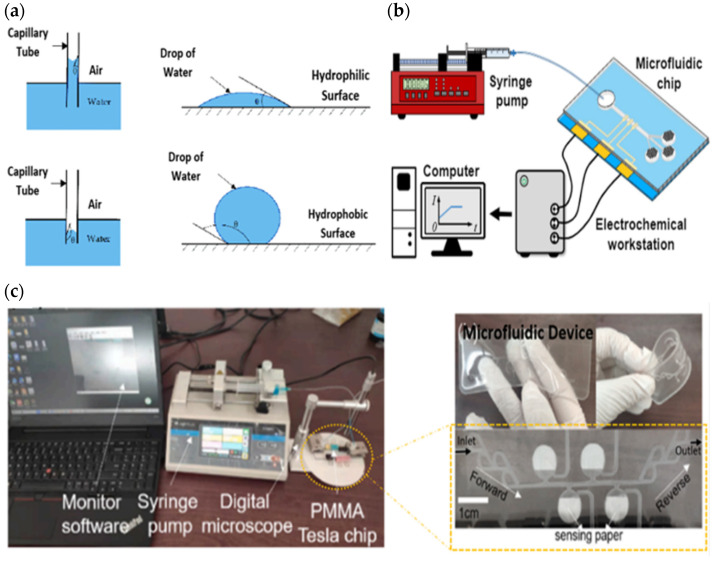
(**a**) Hydrophilic and hydrophobic surface performance [135] (reused with the permission of copyright 2014, John Wiley and Sons). The syringe pump, as the active pump, controls the fluid flow into the hydrophobic PDMS microfluidic chip during an off-body test. (**b**) Schematic diagram [127] and (**c**) an image of the real device involved. Reprinted from [136], copyright (2022), with permission from Elsevier.

A microfluidic device basically has a channel dimension of tens to hundreds of micrometers to reduce sample consumption and miniaturize microscale instruments for portability [126]. It largely uses body fluid samples such as sweat for the point-of-care diagnosis of diseases and certain laboratory tests. A microfluidic device can be sorted into the mechanism of active micropumps [127,128] and passive micropumps [129,130,131,132,133]. An active micropump requires an external power source to guide a continuous fluid flow with an adjustable flow rate into a microchannel. Typically, an electronic pump is used to deliver a solution efficiently with a setup of a steady flow rate at an inlet cavity that is similar to the natural average velocity [127], pressure, and mass flow rates of a biofluid. It is mainly used to enhance the smooth movement of liquid in a hydrophobic based material channel. Hydrophobic surface will increase resistance of capillary action to the fluid flow during passive flow. Its low surface energy makes it hard to wet on the wall surface, resulting in time-consuming sweat collection on a sensor surface [134]. Figure 5a shows that the water level flow in a hydrophobic capillary tube is lower than a hydrophilic one, demonstrating that a hydrophobic surface has high resistance and requires external energy to keep a liquid flowing. Thus, the external pressure exerted on fluid by an applied active micropump (electronic pump) can ignore the weak conditions of the hydrophobic surface. This review highlights this functional pump so that other researchers can improve the performance of active pumps that can be used for both on-body and off-body tests, reducing the time required to implement two different procedures by using a similar active pump. Figure 5b,c demonstrate a syringe pump controlling a solution entering a PDMS microfluidic device during a calibration/off-body test [127].

**Figure 6:**
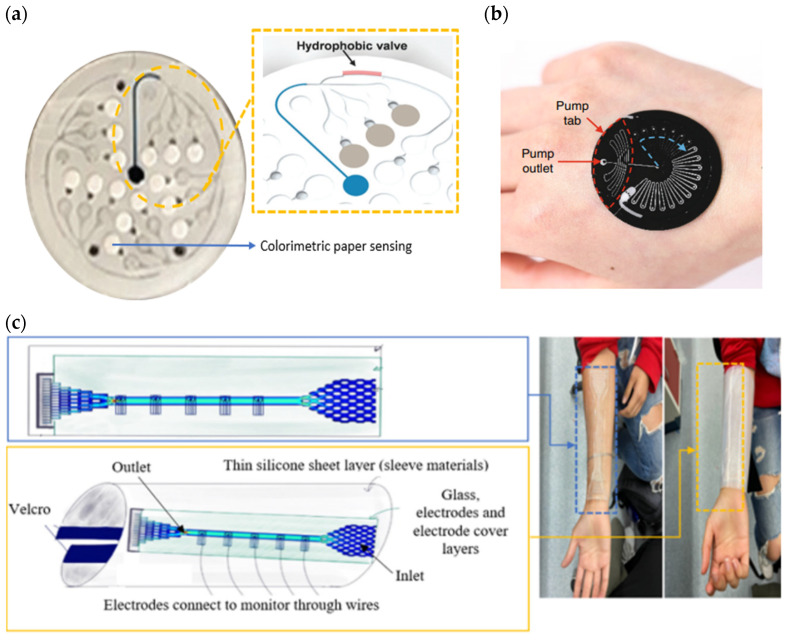
Microfluidic devices with passive micropumps. (**a**) Incorporating hydrophobic valves [138] (reused with the permission of copyright 2018, John Wiley and Sons). (**b**) Incorporating capillary-bursting valves and a soft pinch valve [139]. (**c**) A hydrophobic PDMS microfluidic device that has been bonded into hydrophilic material of glass for passive micropump; the image was taken from https://2021.igem.org/Team:Rochester/Hardware#here2 (accessed on 26 February 2022).

Recent studies have focused on improving fluid flow control in microfluidic devices via passive micropumps due to their simplicity of use and the fact that they do not require an external power source. They usually involve pressure and capillary forces embedded in the device itself to transport sweat secretion into the channel. Incorporating capillary-bursting valves in hydrophobic microfluidic devices allows fluids to flow with high capillary force into the sensor due to the different pressures between the open valves and the microchannel [137,138], as shown in Figure 6a. PDMS’s hydrophobic properties have high resistance to water flow on its surface, whilst adding a valve forces a continuous flow of sweat. It is also able to avoid accumulation, dilution, cross contamination, flow-through mixing effects, and other surface fouling effects. In addition, this valve chamber prevents backflow and reduces sweat evaporation [131]. A microfluidic device design that incorporates valves typically consists of a serpentine (for high complexity shape used to increase the surface area of the fluid reservoir), a soft device, and colorimetric sensing. For example, Reeder et al. developed a colorimetry method of a hydrophobic microfluidic system that combined suction and an outlet pump with soft pinch valves, as shown in Figure 6b [139]. Their device enabled the ejection of sweat to restore the device to its initial empty condition while metering delivery or the extraction of fluids. Besides incorporating valves in a microchannel, fabricating a microfluidic device made of hydrophilic material also enhances a passive micropump. A hydrophilic device does not necessarily require an external force device to effectively allow a biofluid to flow across a channel into a sensor. Otherwise, a microfluidic device based on hydrophobic material can be modified to create a hydrophilic surface (water-loving, promoting high energy surface to wet easily and fast) by plasma treatment (chemical top coatings for surface treatment) between PDMS and glass [132,134,140,141], as shown in Figure 6c. Even if the modified surface of PDMS becomes hydrophilic, the surface will gradually lose its hydrophilicity and be restored to a hydrophobic state within a few minutes [142,143,144]. An alternative approach is to immerse the material’s surface in water immediately after plasma treatment or use solvent extraction to remove uncured polymer chains, which effectively slows or prevents this recovery [145,146].

**Figure 7:**
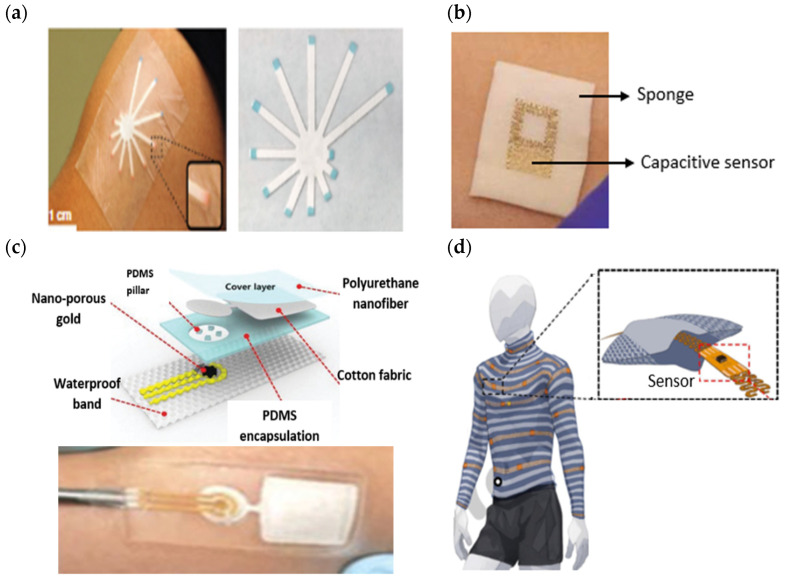
Sweat-based material absorption. (**a**) Filter paper [149]. (**b**) Sponge [147] (reused with the permission of copyright 2014, John Wiley and Sons). (**c**) Cotton fabric. Reprinted with permission from [153]. Copyright 2019 American Chemical Society. (**d**) Textile [113].

Meanwhile, absorbent-based material deals with liquid flow manipulation through the gaps in a porous media substrate that has the advantages of offering water absorption [34,147,148,149] or stimulating sweat secretion [150,151,152]. Adding porous substrates between the sensing component and the skin can prevent the blockage of glands, remove direct contact, and reduce abrasion. Sweat-based material absorption and stimulation are often utilized for purposeless extra energy due to good fluid extraction and inherent capillary action. Sweat-based material absorption regularly employs functional tools such as filter paper [149], sponge [147], cotton [34,153], and textiles [113,148], as shown in Figure 7a–c. They are passive, fluid-driven and capable of manipulating the hydrophobic–hydrophilic surface [151]. This porous substrate of absorption material creates a high capillary pressure that can wick gradually, increasing fluid flow into the sensor. Additionally, it has an excellent water diffusion capability and is readily available, lightweight, low-cost, biodegradable, thin, and easily attached to the skin as it can be miniaturized in its design. Sweat-based substance absorption is useful for individuals who secrete a low sweat rate and can be used in a microfluidic device for effective and quick sample collection. It also can be used as a substrate for colorimetric sensing, as proposed by Jain et al. [149]. They developed an epidermal patch consisting of laminated filter paper patterned into radially arranged fingers with their tip having triggered spots of water-activated dyes, as shown in Figure 7a. Each triggered spot changes color once fully filled at the fingertip of the cellulose paper strip, providing easily identifiable levels of water loss for mapping to personal dehydration levels.

**Figure 8:**
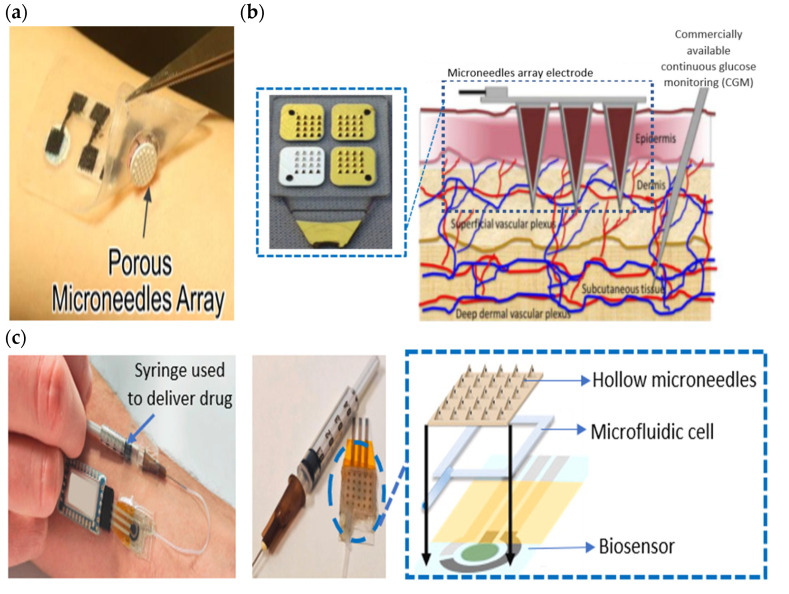
(**a**) The sweat-based material stimulation (hydrogel) mounted with porous microneedles array [161]. (**b**) Illustration of microneedles in the skin layer structures. Reprinted from [158], copyright (2019), with permission from Elsevier. (**c**) Scheme layout of microneedle array component in a single SSD. Reprinted from [159], copyright (2019), with permission from Elsevier.

Sweat-based material stimulation, such as the iontophoresis of pilocarpine [80] and hydrogels [150,151,152], triggers the activation of the sweat gland without the need to force sweat secretion (e.g., performing physical activities, exposure to a hot environment or an emotional regulation effect). Moreover, the lag time of sweat production can be reduced by epidermal iontophoresis [14]. It also offers the flexibility of enabling temporal sweat analysis at various body locations, as well as the capability of producing and controlling sweat induction, making it particularly favorable for use in point of care. Most previous studies and commercially available sweat analysis instruments on sweat stimulation using an iontophoretic technique have specifically designed iontophoretic electrodes with separate methods for sweat induction, collection, and detection [79,154]. Meanwhile, hydrogel is easily cell-cultured and generally biocompatible. It can be made from various materials with different abilities to induce an affinity of sweat volume, for example, alginate, agarose, phosphate-buffer saline (PBS), polyacrylamide, and glucose. Hydrogel can also provide a better sweat-swelling volume than other stimulants and absorbent materials [155]. Sometimes, it can consolidate with a hydrophilic microneedle that can penetrate through the stratum corneum, the top-most layer of dead skin cells, and into the dermal interstitial compartment [156,157], as shown in Figure 8a. Microneedle arrays are minimally invasive because they are small in size and have a low penetrating depth [108]. As a result, they are associated with fewer side effects such as skin irritation, pain, and tissue trauma, and skin micropores recover in less than 24 h with minimal infection and bleeding in the local area [158]. In particular, sweat sampling methods using microneedles involve transdermal electro-osmosis penetration and the extraction of target analytes by a porous microneedle. In the initial procedure, a specific drug is applied to the skin for transdermal injection, delivered to the body over time with a microneedle injected into the target body [159]. It then creates a large capillary pressure, quickly drawing interstitial fluid from the body. However, the microneedle’s fluid drawing potential stops after it fills the sweat ducts. The interstitial fluid (ISF) that is generated through this approach shows a good correlation with venous blood [160].

**Figure 9:**
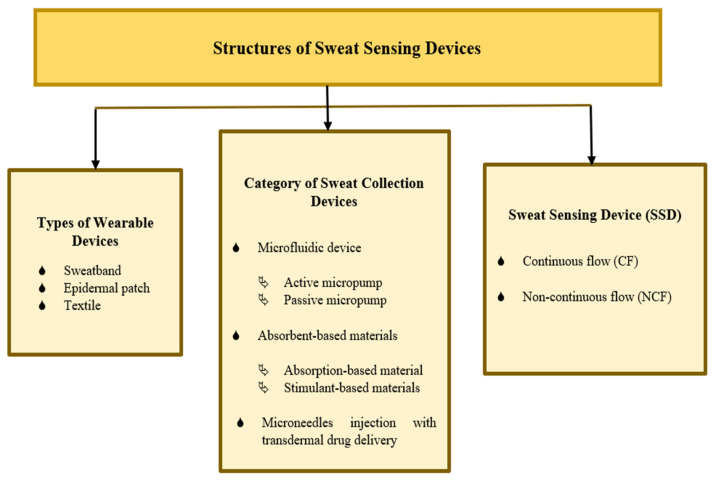
Summary of sweat-sensing device structures.

The combination of a microfluidic device, absorbent-based materials, and microneedle injection demonstrates the best improvements in sweat collection devices, which has a high potential for efficiently transporting a solution in a short time while sharing its combined advantages and overcoming each other’s limitations. Figure 9 shows a summary of various SSD structures, including the types of wearable devices, categories of sweat collection devices, and sweat-sensing devices. There are two main groups for classifying sweat-sensing devices: continuous flow (CF) and non-continuous flow (NCF), based on the presence or absence of an integrated device outlet and real-time measurement. A microfluidic device consists of an outlet, allowing fresh sweat to continuously pass through a sensing area, providing the capability of performing continuous real-time analysis [162,163]. Real-time computation is essential for evaluating precise and valid current sweat analyte concentration withdrawal at a particular time, especially for sweat analysis over a longer period of time [109]. A sensor is commonly used to measure target sweat analytes in real time. CF and NCF SSDs can also be varied in terms of the types of wearable devices and sweat collection devices.

### 3.1. Non-Continuous Flow (NCF)

An SSD for NCF measurement design commonly does not consist of an outlet in a microfluidic device or an unincorporated sensor. SSDs without an outlet do not allow for continuous measurement, leading to pooling and the circulation of a renewal of sweat concentration in a channel [163]. Furthermore, they cannot perform real-time analysis of continuous sweat samples without an integrated sensor [164]. Therefore, they are best suited for clinical diagnosis applications that necessitate a small volume of sweat. This small volume is enough to fill the depth channel of a microfluidic device without an outlet opening in order to minimize the mixing of new and old sweat. In general, iontophoresis often generates a small amount of sweat, significantly less than when undergoing vigorous physical activity. Thus, most NCF SSDs exploit chemical stimulation to produce an adequate low sweat volume for measurement. In addition, the minimal volume of sweat produced due to iontophoresis is enough for detecting disease reliably [165]. This section addresses SSDs of an NCF type regarding their model design, features, performance, and device function for exploring SSDs used in different sweat collection tools.

**Figure 10:**
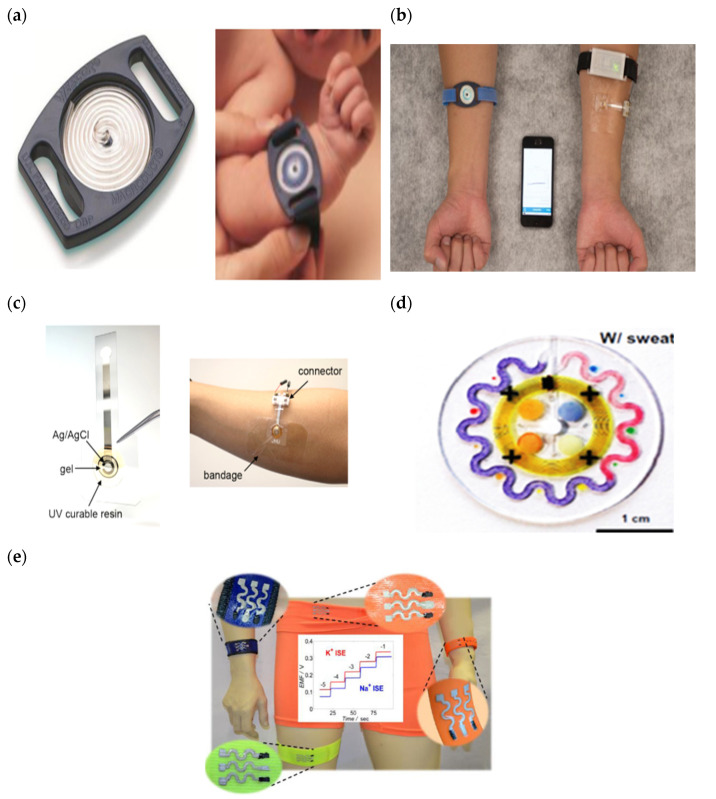
Sweat-sensing devices for non-continuous flow (**a**) Macroduct^®^; image was taken from https://www.elitechgroup.com (accessed on 3 February 2022) (**b**) NCF devices in diagnosing CF using iontophoresis of pilocarpine with a conventional sweat test (left side) and a wearable sweat sensor in real-time (right side). Reprinted from [167], copyright (2018), with permission from Elsevier. (**c**) The wearable patch of NCF adopted hydrogel. Reprinted from [168], copyright (2017), with permission from Elsevier. (**d**) The wearable patch of NCF consists of a colorimetric, microfluidic device and hydrogel for physical exercise analysis: from [174]. Reprinted with permission from the American Association for the Advancement of Science. (**e**) A wearable NCF textile for fitness performance [176] (reused with the permission of copyright 2016, John Wiley and Sons).

ELITech groups introduced sweat testing for detecting cystic fibrosis using three separate procedures, including sweat stimulation, collection, and analysis [166]. Their commercial sweat stimulating device (Webster Sweat Inducer Model 3700) is able to conduct the iontophoresis of pilogel disks (pilocarpine) at electrodes within 20 s. The electrodes are removed after iontophoresis, and a wearable Macroduct^®^ device is applied. The continuous sweat secretion creates hydraulic pressure that forces fluid accumulation on the skin through spiral microtubing into the Macroduct^®^ device. The transparent spiral microtubing is turned blue when the fluid fills it, as it contacts with a small amount of blue water-soluble dye applied to its collection surface, as shown in Figure 10a. This condition allows an easy assessment of the sweat volume level at any time during collection. The spiral tubing is disconnected from the Macroduct^®^ device when the blue color indicator reaches the tube’s end. The sample collection in the spiral tubing is then measured using the sweat-check analyzer device for chloride analysis. The sweat measurement is not done in real time because different processes are implemented at different time intervals and do not involve a sensor; hence, this device is classified as NCF. Moreover, the Macroduct^®^ device features a sealed aperture for the outlet.

Choi et al. developed a sweat testing device for cystic fibrosis using two methods, namely a conventional laboratory test (a similar procedure to ELITech group’s) and a wearable sweat sensor [167], as shown in Figure 10b. Their wearable sweat sensors showed high correlation results with the standard clinical method over a wide chloride concentration range. In addition, the wearable sweat sensor could detect and quantify a smaller sweat volume of 13.1 ± 11.4 μL compared to the conventional laboratory testing volume of 15 μL. Furthermore, utilizing a sensor ensured real-time sweat chloride measurement, while the conventional laboratory test performed sweat collection and measurement separately. Both devices are also considered NCF because they do not have an outlet opening in the micro-collector for continuous measurement, and the conventional laboratory test also does not measure in real time.

Choi et al., in another study of a sweat-sensing device, proposed a wearable sweat sensor with hydrogel to diagnose cystic fibrosis disease and analyze an athlete’s performance during exercise on a stationary bike, as shown in Figure 10c [168]. Their non-continuous wearable sensor consisted of a sodium sensor, PET film (hydrophilic polymer material), salt bridges (laser-drilled hole), hydrogel, and an adhesive bandage. A reference electrode consisted of an agarose gel containing 1 M potassium chloride, which was sealed with an ultraviolet curable resin to prevent the evaporation of the reference solution in the hydrogel. Meanwhile, a working electrode was attached on the bottom side of the PET film that directly contacted the skin when attached to the body. The salt bridge provided an ionic path between the reference and test solutions for accurate measurement. Since the film was made of hydrophilic material and did not have absorptive capabilities, it was ideal for allowing the sample to flow into the sensor for fast sweat measurement [169]. However, their device might result in a mix of new and old sweat over a prolonged measurement duration because it does not comprise an outlet.

An epidermal patch that involved a combination of cotton (absorption-based material), hydrogel (stimulant-based materials), and microneedles for interstitial fluid (ISF) evaluation was reported by Kusama et al. [161]. Their porous microneedles (PMN) induced minimal skin irritation and pain because the microneedle length was designed at 250 μm to prevent them from reaching the sensory nerves in the dermis layer [170]. They adopted transdermal drug delivery by passing a direct current through pig skin while also incorporating PMNs to improve iontophoresis with charged hydrogel, as shown in Figure 8a. As a result, it reduced transdermal resistance and created electroosmotic flow, aiding glucose penetration for the efficient drug delivery and extraction used for ISF analysis. After 1 h, a 40 μL portion of the glucose solution in the ISF was extracted between the PMN and a cathode, which was then analyzed using a glucose assay kit. 

NCF SSDs are also acceptable when assessing fitness performance that continually stimulates sweat by extreme exercise intended to produce an excessive sample that affects the circulation of new and old sweat in a microchannel without an outlet. In this case, they require an indicator in the form of a fluid reservoir to determine complete filling in the microfluidic channel, for example, colorimetry [171,172], which uses a color benchmark. The colorimetric sensing method manipulates color change by clearly observing a threshold indicator of fluid filling to provide a basic warning of the microfluidic device channel being full in order to stop measurement [149]. It is a simple technique that uses the human eye to inspect different color changes to compare and determine a few analytes in a reservoir tool while displaying the fluid mark load. It is able to evaluate both a quantitative and qualitative analysis for the presence of the target analytes [173].

Kok et al. used colorimetric sensing to measure the analytes present in sweat using nanofibrous sensors [174], as shown in Figure 10d. Specific chromogenic reagents were added for detecting the target analytes, and it was located on filter paper and inserted into containment reservoirs. Hydrogel (made of cobalt chloride dissolved in polyhydroxyethylmethacrylate) was also included in the spiral channels. A thin PDMS layer was sealed between the bonding of the spiral microfluidic channels, containment reservoirs, and wireless thin electronic systems. In addition, the final design of this device formation included openings created to define the inlets for sweat collection but not the outlets. It was then covered with double-sided adhesive tape on the bottom device, which kept it attached to the skin. This described colorimetry microfluidic device design could retain almost 50 μL of sweat, corresponding to an effective working time of 1 to 6 h of exercise depending on the rate of sweat loss. The opening inlet diameter was 3 mm, corresponding to 10 sweat glands [175].

Textile substrate-based sensors can be categorized as NCF. Parilla et al. tested a textile as a wearable device and a substrate of sweat-based material absorption while embedding a sensor during prolonged exercise, as shown in Figure 10e [176]. The analyte concentrations of the sweat were absorbed by fabric with sensors attached during physical activity to provide a valuable indicator of dehydration [177]. Their design was attractive as it was a highly stretchable textile, resilient, and repeatable for transducing the potential response of analytes under extreme mechanical stress after conducting a strain test, bending, crumpling, and washing assessment. Moreover, the modification sensor materials (polyurethane and carbon nanotube) used were shown to minimize unwanted inflammation when the electrodes were in contact directly with the skin. This result was similar to Yun et al. [178]. However, there is concern about mixing the concentration of fresh and old sweat secretions during dynamic measurement in this textile absorbent. Adding a microfluidic device with an outlet for a textile substrate-based sensor can solve this problem while providing real-time monitoring [162], but it must ensure the sensor’s reusability for analysis through proper microfluidic design and possible self-cleaning ability [179].

### 3.2. Continuous Flow (CF)

A CF SSD measures a dynamic sweat sample concentration for a long time without blending old perspiration while offering real-time analysis. It comprises channel links that effectively enhance fluid movement in and out. Even though microchannels of a small size and depth are used, a large volume of sweat can be dealt with using CF SSDs. Previous sweat is removed via an outlet, while current fluid is drawn to substitute an already measured solution that passes through an embedded sensor. Therefore, it leads to the production of a high volume of perspiration when continuously secreted fluid flows in a microfluidic channel, and it does not mix the renewed sweat with old sweat as it contains an outlet. The CF SSD is primarily suitable for physical exercise to monitor the sweat rate and target analytes in sweat from subjects exercising for a prolonged time. The indication of the body’s overall liquid and analyte loss during exercise is a possible way for giving athletes a heads-up when they may be pushing themselves too hard [99]. In addition, CF can also incorporate sweat sensors to track the flow rate of sweat and report how much a person is sweating.

The electronic micropump serves as an artificial or assisting pump in running the function of transporting a solution passed through a microchannel and sensor continuously. Remarkably, it is usually used during the calibration of the sweat flow rate for a developed microfluidic device and the validation of sensor reliability. Cheng et al. tested the performance of their developed PPMA microfluidic device embedded with an electrochemical sensor for real-time detection of the flow rate of sweat secretion in a microchannel. They utilized a sodium chloride (NaCl) solution as a sweat mimic as the driving fluid in a hydrophobic microfluidic device by connecting the syringe pump at the inlet with varying the predefined input of flow rates of 0.2–1.2 μL/min [127]. The transport of the old sweat solution was also aided by the capillary force of the fluid filling and followed the spontaneous evaporation effect of the micropores on three outlet cavities. Different flow rates were applied to determine the varying intensities of physical activity in perspiration for current sweat measurement. The whole system of the experimental result for CF using a syringe pump was transferred by an electrochemical workstation into a computer for further analysis, as displayed in Figure 5b.

**Figure 11:**
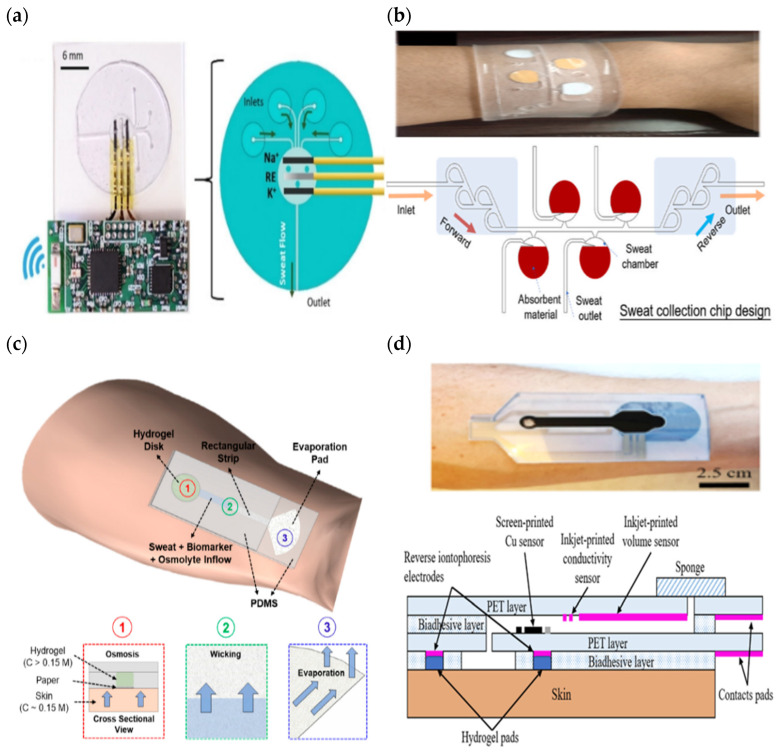
Sweat-sensing device for continuous flow (**a**) The epidermal patch of hydrophobic microfluidic device [180] (reused with the permission of copyright 2018, John Wiley and Sons) (**b**) The epidermal patch of hydrophilic microfluidic device mounted with absorbent material. Reprinted from [136], copyright (2022), with permission from Elsevier. (**c**) The wearable patch of the CF of hydrophobic microfluidic device embedded with paper and hydrogel to measure sweat lactate [182]. (**d**) The epidermal patch of multiplexed sensors (volume sensor and copper sensor) mounted with conductive hydrogel, a sweat-absorbent sponge at the outlet, and a hydrophilic microfluidic device CF. Reprinted from [184], copyright (2022), with permission from Elsevier.

In addition, Sempionatto et al. developed a sweat sensor embedded with a hydrophobic microfluidic device (that comprised four inlets and one outlet) and interfaced with a wireless electrical printed circuit board capable of on-site signal conditioning, analysis, and transmission, as shown in Figure 11a [180]. Multiple inlets ensured that an adequate amount of sweat was efficiently delivered toward a sensor for the continuous monitoring of potassium and sodium during an on-body test (such as cycling). In their design, they ignored the hydrophobic channel effect. Instead, they considered the human body capable of triggering natural sweat pumping via continuous biological sweat secretion during prolonged exercise, which forces biofluid flow across the microchannel. During the calibration test, an Instech pump was used to inject 0.1–200 mM of the testing solution into a microfluidic device with three repetitions of 20 mL injections to validate the functionality of the developed microfluidic device for continuous fluid flow. Meanwhile, certain output parameters, such as velocity, streamlines, pressure, and pressure associated with streamlines, were examined in the theoretical simulation.

The International Genetically Engineered Machine (iGEM) teams designed a microfluidic device made of PDMS for developing micro-reservoir chips (containing micropillars) and glass slides (hydrophilic) for use in closing the channels, as shown in Figure 6c [163]. Hydrophilic properties and micropillars function as a passive pump to increase the surface area and forces of wicking for sweat filling the channel into a biosensor and then to exit channels. They validated the performance of their generated microfluidic device through both experimentation and simulation (COMSOL software). When the flow rate at the inlet was set to 0.1 mL/s, the velocity of fluid flow in the center device (where the biosensor was located) was 8.11 mm/s for the experimental test, which was slightly slower than the simulation of 10 mm/s. The average sweat rate at rest when chemical stimulation was induced was 0.067 m/s. When the flow rate at the inlet was 0.067 m/s, the exhibited the volumetric flow rate was 0.13 m/s for the simulation and 0.12 m/s for the prototype. Thus, it indicated that their developed device was reliable as it had a high accuracy and almost matched the actual fluid flow rates of the theoretical simulation. They added a durable silicone sheet layer and a semi-permeable thin film as a wearable sleeve to attach their device to the patient’s skin to permit vapor and prevent bacterial transmission [181].

On the other hand, Shi et al. developed a wristband in the form of a sensor to detect pH and glucose in sweat with an integrated hydrophobic microfluidic device consisting of Tesla valves and absorbent pads (filter paper) [136] as shown in Figure 11b. The PDMS channel was treated with a polyvinylpyrrolidone (PVP) ethanol solution and silica gel to create a hydrophilic surface and increase the capillary force for sweat collection. In addition, micro-valves were added at the inlet and outlet, providing one way flow and a continuous measurement of the testing solution. The presence of valves created a pressure difference that drove the fluid’s forces, with a boost flowing in the forward direction and hindering flow in the backflow path. They also validated the design of the micro-valves using simulation and off-body experiments with a syringe pump and digital microscope (as demonstrated in Figure 5c). Their study showed that the valves in the microfluidic device provided better performance at lower flow rates ranging from 0.05–0.3 m/s for a total volume chamber of 200 μL.

Saha et al. developed a wearable sensor that combines hydrogel plates, capillaries, and evaporation action using a paper microfluidic device, as shown in Figure 11c [182]. The hydrogel disc extracts sweat via osmosis and delivers it to the paper during passive sweating (when at rest). In the absence of hydrogel, the paper can capture sweat during active sweating (exercise). The withdrawn sweat is routed via a paper microchannel and evaporated at a pad to keep up the continuous inflow of sweat. In their observation, they found that the hydrogel glucose solution was the best in delivering long-term, compared to both the PBS hydrogel and without hydrogel (replaced with PDMS disk). Based on their result, the 4 M hydrogel glucose produced an average sweat volume of 1.8 µL after 2 h of testing during rest and correlated with the results reported earlier by Bhide et al. [183]. For active exercise, 4 M hydrogel glucose produced an average sweat volume of 2.7 µL (medium-intensity exercise), 1.6 µL (post-medium-intensity exercise), 11.3 µL (high-intensity exercise), and 1.6 µL (post-high-intensity exercise) of fluid on the paper after 2 h. 

SSDs of a CF type involve all types of sweat collection devices, enabling the resolution of several common issues in SSD development, such as low sample extraction, real-time measurements, continuous monitoring, and the prevention of the mixing of refreshed sweat. Yang et al. adopted a hydrogel pad in contact with the skin while integrating PET layers as a hydrophilic microfluidic chamber, and an outlet was added in the form of a paper-based sponge [184], as shown in Figure 11d. A conductive hydrogel stimulated sweat secretion and allowed the solution to fill the surface of the copper sensor in less than 7 min. The inkjet-printed layer of PET was briefly treated with atmospheric plasma to increase its hydrophilicity. However, the hydrophilicity of the surface was lost after 23 days. Meanwhile, the sponge improved fluid sample movement by using a highly absorbent liquid and enabling evaporation to empty the microchamber. In their SSD, a sweat sensor and a screen-printed copper sensor were attached to the microchannel’s wall to measure the sweat rate normalization, real-time sweat analysis, and signal inducing (when the channel was completely filled). The depth size of the total volume sample collection in the microfluidic device was about 27 μL.

## 4. Recent Advances of SSDs: Optimal in Designs, Functionalities, and Performance

An SSD’s main components, including sweat collection devices, sensors, and electronic devices, are key parts that can be used to optimize its system designs, functionality, and performance. The enhancement features of current sweat collection devices are portable chemical sweat stimulation, optimizing their design, which allows fast sweat collection, repeatability, the prevention of skin discomfort, maximizing validation methods for reliable measurement, and high resilience. Meanwhile, the latest developed sensors have improved their efficiency by selecting the best materials that give excellent sensing performance (e.g., stability, sensitivity, repeatability, and robustness), highlighting the best conditions to employ optical and electrochemical sensing and utilizing multiple sensors. This section also discusses the recent development of electronic devices that covers miniaturized, battery-free systems, wireless communication, and machine learning. These proposed advanced features and their explanations are described in detail in their respective subsections.

### 4.1. Sweat Collection Device

Adopting pharmacologic agents of sweat-stimulation such as hydrogel and pilocarpine can induce the skin to generate local sweat without requiring physical activity. The iontophoresis of pilocarpine can produce a sufficiently small amount of perspiration within the range of 15–100 μL [185]. Typically, the minimum volume capacity that a chemical sensor can react to and measure is at least 10 to 100 microliters [186]. In addition, this sweat stimulation can also be used to increase the biofluid for an individual that produces a low volume of sweat secretion during exercise. Moreover, the average sweat rate is approximately$\;1–15\;{µL\;\min}^{-1}$ during active secretion [187,188], which slows fluid generation. Therefore, the use of pilocarpine/hydrogel is advocated for stimulating eccrine glands to elevate perspiration levels quickly for exercise and rest. However, most conventional pilocarpine iontophoresis methods are commonly utilized separate processes for collecting sweat samples that are inapplicable outside of the laboratory. Moreover, this process exposes electrical power that can cause skin burning, relying on expensive laboratory equipment and both bulky electrodes and benchtop analyzers [99,102]. Thus, adding a sensor while using this method can provide the dynamic real-time monitoring of target biomarkers on the spot. Kim et al. developed an SSD that can simultaneously implement the iontophoresis of pilocarpine and the real-time measurement of the continuous monitoring of sweat glucose and alcohol [106]. Their epidermal device consisted of a sensor and anode and cathode electrodes. An anode delivers transdermal pilocarpine while a cathode extracts interstitial fluid (ISF) to realize sweat stimulation in a portable SSD. The wireless iontophoresis of pilocarpine can prevent skin burning and irritation during the prolonged monitoring of biomarkers due to its low current and by applying agarose gel.

Employing multiple sweat collection devices in a single SSD can efficiently accumulate a sufficient volume of sweat in a short duration and is able to provide fast hydration monitoring. Gunatilake et al. demonstrated a signal detection time readout of 4 min for lactate and 6 min for glucose using microfluidic paper-based analytical devices by incorporating a hydrogel of alginate-based materials and a colorimetric biosensor [189]. In addition, Alizadeh took 7–10 min for a hydration monitoring application using hydrogel that induced more sweat secretion while utilizing hydrophilic glass wicking in a microfluidic device [152]. In contrast, Nyein et al. only applied a single sweat collector in a microfluidic device which took 30–45 min [128]. However, the period for fitness performance analysis does not mostly depend on the number of sweat collection devices used because it can be flexible when continuously measuring at the user’s preferred target time for estimating the loss of the electrolyte composition. The assembly of various sweat collection tools is primarily concentrated on supporting and maximizing the functions of each other. Incorporating a microfluidic device can hinder the problem of contamination, fast evaporation that reduces the sample collection, and an inevitable blend of renewal sweat concentration [190,191]. In addition, the introduction of a hydrophilic channel can promote capillary pressure, wall adhesion, and hydrostatic forces for the sweat flowing optimally in and out of the chamber. Ideally, a paper-based microfluidic patch could be a viable option to eliminate the direct contact with sensors that causes abrasion, avoid blocking the breathability of glands on the skin, and avoid backflow by increasing the pressure at the inlet during long-time monitoring [192]. Moreover, it facilitates continuous flow collection by transporting the sweat composition in liquid-filled paper channels, and it can also reduce the mixing of old and new sweat [193].

Microneedle transdermal injections can introduce new microbiomes from the surroundings into the skin’s micropores. Although research has demonstrated that skin barrier function can recover the micropores within a few hours [194], utilizing wearable microneedles increases the risk of infection because bacteria can circulate through open micropores on the skin [195]. Moreover, hollow microneedles may fail and shatter due to additional compression, shear stress, excessive motion, the inherent discrepancy in complexion, or any other related pressures [196]. So, standard operating protocols for the use of microneedles are necessary for implementing proper clinical practice. Several mechanical and biological factors of evaluation approaches could be performed in vitro and in vivo to precisely assess possible risks and examine the safety of skin contact with innovative devices, especially when microneedles are used [197,198].

The repeatability of a microfluidic device is vital to ensure it can be frequently reusable, more practicable, prevent the waste of equipment, and avoid relying on disposable products even if they are the cheapest. Yang et al. introduced a reusable microfluidic device that integrated a high selectivity of concurrently detecting lactate and uric acid [199]. Their microfluidic device is portable, small in size, and has lower sample consumption, showing a good design for application prospects in clinical testing and personalized healthcare. Another study developed a microfluidic device that can be repeatedly reused by requiring its chips to be flushed with deionized water to remove the previous biofluid-measured ionic background [34]. Their microfluidic device collects the sweat volume with a small depth size to reduce time-consuming fluid flow. Their wearable microfluidic device is integrated with optimally modified sodium sensors that can measure several applications, such as fitness performance and diagnose cystic fibrosis.

Adding multiple inlet openings to a microfluidic device increases the exposed skin region that releases sweat into the channels while reducing time-consuming sweat secretion that depends on the accumulation in one hole. Xu et al. designed a microfluidic device consisting of eight inlets to reach a reservoir channel at the surface contact of a uric acid electrode sensing interface through the capillary effect [187]. During off-body test, they tested the performance of the microfluidic device by inserting a dye solution with a flow rate of $15\;{\mu L\;\min}^{-1}$ at the inlet. The time required to fill the microfluidic reservoir completely was 166 s. They switched the concentration of the solution between 0–80 μM with a flow rate of $15\;{\mu L\;\min}^{-1}$, while the well-mixed renewal sweat could be used to determine the renewal time, which came out at 4.68 min. Meanwhile, adding multiple outlet openings in a microchannel could also improve passive pump performance through the spontaneous evaporation effect. Cheng et al. developed a wearable sweat sensor consisting of three micropores of the outlet with an inlet cavity [127]. The old sweat solution was quickly transported out by the fluid filling induced by capillary force and followed the spontaneous evaporation effect of micropores at three outlet cavities.

A simulation must be conducted for the fluid flow rate in a microfluidic device before developing its prototype. This is to determine the future channel size and what material properties of the wall are used to provide sufficient pressure without applying external energy. Moreover, a fluid simulation can also be used to mimic a real experiment for the validation of the channel performance of a microfluidic device by defining the average velocity of biological sweat secretion at the inlet. In the experiment, a flow rate sensor must be integrated with a microfluidic device to accurately measure the fluid flow rate. A high correspondence of a good flow rate and short-time results between the fluid simulation and experimental results exhibit the best verification of the effective working of microfluidic device channels. Nyein et al. [128] and the iGEM teams [163] realized both tests, varying the flow rates and solution concentrations. Hence, they had the advantage of exploring and gaining more information about the performance and capability of their device through a variety of changing channel sizes, shapes, and materials via theoretical simulation while saving time and money.

Table 1 shows other more recently developed SSDs with some features related to sweat collection device design. Most of them are CF SSDs that are integrated with sensors for real-time measurement and consist of an outlet in a microfluidic device. In addition, their developed microfluidic device promotes repeatability even though the wearable device is a single-use, disposable epidermal patch. This patch could be replaced with a new patch to allow the sweat collection device to be reused. Moreover, several of them incorporated microchannels with three, six, and eight inlets. Suction pumps and valves were also added to their microfluidic systems to control and increase fluid flow movement. Furthermore, the proposed microfluidic device’s validation method was tested, including at least off-body test (calibration) and on-body tests to compare both evaluation performances. In addition, a few of them included a simulation to test the mechanical performance [14,200] as well as the performance in terms of fluid flow [201,202]. Mechanical testing is required to establish the level of flexibility and robustness of the microfluidic device in terms of bending, stretching, and twisting to identify which types of extreme activities they are appropriate for use in. For textile SSDs, it is essential to undergo a process of cleaning and drying for reusability. However, its design should be unaffected by the process, allowing it to retain its functionality.

### 4.2. Sensor

Maintaining a high level of stability in measurement while continuously monitoring a sensor’s reliability is challenging. In addition, a high degree of contact between an SSD and the skin can create noise artifacts caused by underlying skin strain or movement. Thus, a great resiliency and robustness of the sensor during extreme physical exercise are essential to enhance the reliability of sweat measurement with minimized dynamic motion artifacts [205]. These mechanical characteristics can maintain a good stability of the sensor’s potential, current, or impedance of readable signals for prolonged monitoring even in fluctuating ion concentrations of analytes. The optimization of a surface electrode with polyurethane [176,206], carbon nanotubes [33,207,208], Nafion [207,209], or Ecoflex [210,211] on the solid-contact coating has commonly maintained the contact of electrical conductivity and further high resistance to mechanical stress to ensure strong adherence to conventional substrates. Furthermore, these materials’ properties generally include metallic conductivity, high tensile strength, high elasticity, thermal stability, high chemical inertness, and small sizes that are favorable for electrochemical sensor performance [212]. Moreover, with improved solid contact membrane features of electrode sensors based on these materials, the energy generated from mechanical strain can be absorbed, rearranged, and accommodated without deforming, debonding, fracturing, or distorting the electrodes, showing a significant advantage [213]. Hence, the use of modified chemical sensors on the surface of electrodes is essential for long-term measurement, high durability, and stability during extreme movement conditions of physical exercise on an SSD. In the meantime, these fabricated materials can minimize unwanted inflammation, fouling, and other adverse physiological effects. 

Recently, current state-of-the-art sweat sensor electrodes have frequently been generated from SC-ISEs. Two layers are required: a solid-contact (SC) layer and an ion-selective membrane (ISM). Specifically, the SC layer acts as an ion-to-electron transducer, while the ISM layer acts as an ion recognizer. Introducing material for coating a solid-contact (SC) layer on the first layer before applying an ion-selective membrane (ISM) to the surface of the working electrode (WE) and reference electrode (RE) can also improve a sensor’s sensitivity to detect a specific analyte. The materials most often used in SC layers are poly-3,4-ethylenedioxythiophene (PEDOT) [128,214,215], polyaniline (PANI) [53], Prussian-Blue (PB) [48], Chitosan/Prussian Blue Nanocomposite (ChPBN) [216], and Poly(vinyl acetate)/inorganic salts (PVA/KCl) [100], poly(3-octylthiophene) (POT) [217]. These materials have exhibited the promising features of good sensitivity, high selectivity, and consistent stability in terms of the measurement of electrochemical sensors. PEDOT is a popular SC layer that has frequently been coated on electrode sensors and can be utilized to detect a wide range of sweat biomarkers. PEDOT can be made more effective and more diverse by mixing it with various chemicals such as the synthesis of PEDOT/PB, PEDOT/KCl, PEDOT(1-Ethyl-3-methylimidazoliumbis(trifluoromethylsulfonyl)imide) as PEDOT[emim][NTf2]), PEDOT(1-ethyl-3-methylimidazolium tris(pentafluoroethyl) trifluorophosphate) as PEDOT[emim][FAP]), and PEDOT Polystyrene Sulfonate (PEDOT/PSS),. Matzeu et al. tested the performance of four different chemical solutions of PEDOT, namely as PEDOT(KCl), PEDOT[emim][FAP], PEDOT[emim][NTf2], and PEDOT/PB in detecting sodium ions [34]. All the PEDOT SC-layers that they grew showed an average sensitivity of slopes near the sub-Nernstian of the calibration slope within a concentration range of $10^{-4}-10^{-1\;}$M. They showed that PEDOT[emim][NTf2]) was the best PEDOT SC-layer because of its smallest significance in the standard deviation for the calibration curves of the slope’s offset sensitivity. This SC layer material could give good repeatability and a long life span within 5 weeks, according to Alizadeh et al. [152], and 6 weeks, according to Zuliani et al. [217]. A modified electrode surface with a solid-contact layer of PEDOT/PSS membrane could also give long-term repeatability and stable responses at various physiological temperatures [218]. Wei Gao et al. developed a sodium sensor with reproducibility and long-term stability for at least four weeks by incorporating an SC layer of a PEDOT/PSS membrane on the WE and a CNT membrane on the RE [219].

Particularly, PANI coating has been applied in pH sensors to optimize the analytical performance, including good repeatability and a sensitivity range for pH measurement between 4 and 7 to detect acidic sweat [46,220]. Meanwhile, PB and chitosan are widely used in the fabrication of glucose and lactate sensors, according to a few studies [221,222]. Bandodkar et al. reported that their epidermal tattoo demonstrated an excellent high-detection potential of sweat glucose when a transducer sensor was modified with Prussian-Blue, which exhibited a better performance compare Gluco-Watch, which could lead to compromised selectivity [48]. Moreover, PB is a redox molecule commonly used to modify electrodes to increase the upper detection limit of lactate sensors [223,224,225]. In addition, combining several SC layers, such as ChPBN, can provide a wide range of detection for target sweat biomarkers. As an example, Tanusree et al. detected the presence of sodium ions with good potential sensitivity that a near-Nernstian response of 58 mV for sodium in a wide linear range, i.e., $10^{-7}-1\;$M in a sodium phosphate buffer (NaPB) [216]. Furthermore, their modified electrode provided an excellent stability response with the smallest potential drift of 3.61 × 10^−4^ µV/s, showing a small to almost negligible potential drift, stabilizing the abnormal potential response and increasing the life span effectiveness. Furthermore, the highly porous structure of the ChPBN layer provided a high interfacial area that enhanced the effectiveness of the ion sensing and induced greater selectivity ($10^{3}–10^{4\;}$times higher) towards sodium ions. In summary, most of the proposed SC layer materials used for modifying the ISE sensor surface have promoted good stability and repeatability for long-term use. As a result, there was no ionophore leaking from the ISE into the solution; thus, good sensitivity and selectivity were maintained when the SC layer was introduced. This was because ISM components have a lower solubility due to their improved lipophilicity. This SC also provided more resistance to biofouling, which is advantageous for practical measurements at high concentrations. 

Most ISEs with antibiotic ionophore coatings provide high target biomarker selectivity. For instance, valinomycin ISE has an inherent permeability specific to the recognition of potassium ions, allowing the membrane to detect them selectively [210,226]. However, valinomycin contains biological toxicity that has been often ignored and could be a daunting challenge. A potassium-ion lattice intercalation (PILI) [227,228,229] can be used to replace valinomycin to solve this problem. This is because PILI can potentially form SC-ISE-based battery materials with no need for an ISM coating for sensing potassium ions potentiometrically. PILI can also serve as SC-ISEs with a single-piece structure that is already present with directly mixed SC and ISM for ion-response realization. Thus, this SC layer is capable of both ion recognition and ion-to-electron transduction functions. Moreover, it can prevent the creation of a water layer at the SC/ISM phase boundary as well as prevent the leaking of ISM components, both of which are caused by the ISM. This concept comes from the SC-ISE based on Li-ion battery materials as an SC layer without the use of ISM, wherein the proposed lithium manganese oxide ($LiMn_{2}O_{4}$) electrode exhibits a Nernstian response toward lithium-ion sensing in human blood serum solution [230]. Their study highlighted non-ISM-based SC-ISEs for potentiometric ion sensing, which displayed a comparable sensitivity, selectivity, stability, and linear range to conventional SC-ISEs incorporating ISMs.

Utilizing optical sensing in SSDs enables the provision of a light delivery indicator for the easy visualization and analysis of present particular sweat biomarkers with the naked eye. Colorimetric sensors [191] and fluorescent sensors [231] are the forms of optical sensing most found in recent SSDs. Kim et al. reported a soft, thin colorimetric patch mounted with a microfluidic device that consists of passive valving [112]. Flowing sweat in their colorimetry sensing channel changed the color indicators depending on the loss of iron, zinc, calcium, and vitamin C nutrients. Their device then triggered the delivery of similar amounts of these nutrients with blood transdermally after the sweat secretion filled the depths of a micro-reservoir channel. Most colorimetric sensing applies a simple design without a required electronic device, wireless communication, soft, flexible strength, thin structure, and non-irritating interaction with the epidermis [110,172]. In particular, optical sensors encapsulate specific reagents to produce measurable changes in the optical wavelength for visual information when reacting with the target sweat biomarker. For example, the chemical reaction and absorbance of sweat analyte concentrations by reagent substances in a colorimetric sensor can change color when presenting a specific biomarker [232]. Meanwhile, a fluorescent sensor contains an active chemical fluorescent dye to detect the presence of target analyte concentration changes by responding to a fluorescence intensity change [73]. This sensing method uses optical modules containing LEDs, filters, and a camera to make visible fluorescent probes that detect biomarker concentrations [231].

Recently, wearable sweat optical sensors that function with a smartphone to benefit from technology by capturing and processing images, data analysis, data transfer, data storage, and cloud software systems have been developed [233]. Ardalan et al. developed an epidermal patch with fluorescent sensing based on a smartphone that included the multi-sensing of a wide range of sweat biomarkers in real time, including sweat rate, glucose, lactate, chloride, and pH [234]. In their studies, paper discs with fluorogenic reagents were equipped as sensors, threads in the microchannels retrieved the sweat fluid and conveyed it to the paper sensor, and transparent medical-grade adhesives were used to keep the substrates (paper and thread) in contact with the skin. In brief, an optical sensing device is more appropriate for measuring pH because the results reflect color-changing that is easily observed in color indicator analysis and for multiple analyte measurements that more than three sensors can be integrated [110,172,191,232]. Additionally, this mostly exploits the serpentine channels that store optical assay reagents to increase the surface area of the microfluidic device to store and analyze sweat. In addition, there is no need for a power supply to run the entire system and analyze biomarkers when it is integrated with optical sensing [112].

However, optical sensors perform limited monitoring of sweat sensing in discontinuous flow mode. Thus, adopting an electrochemical sensor into an SSD enables the measuring of target analytes in continuous or discontinuous flow modes. Continuous flow measurement can be set by adding the feature of the outlet channel to a microfluidic device with the integration of a chemical sensor, while discontinuous measurement is the opposite of this design. Discontinuous flow measurement involves the conventional iontophoresis test (Macroduct^®^ system), which is implemented in a separate procedure from sweat stimulation, sample collection, and sweat analyzing. Moreover, an electrochemical sensor has a transducer to detect the target analyte concentration in sweat and convert this chemical reaction into a readable electrical signal (current/voltage/impedance) for amplification and data processing [235]. Amplification is required to increase the readability of the original signal state for such a small concentration unit of the electrochemical signal. The concentration of analytes in sweat is too small, it being in millimolar [236] and micromolar units. The analyzing of readable signals of analytes present in various concentrations of dynamic sweat in real time can be implemented using the electrochemical signal measurement technique. Electrochemical impedance spectroscopy (EIS) [216,237], potentiometric [211,215], and cyclic voltammetry (CV) [238,239] are widely applied techniques in detecting and computing analytes in sweat using electrochemical sensors. Before being performed in sweat analysis on the body, an electrochemical sensor requires a validation and calibration test to establish a reliable electrode. However, an optical sensor can only be used once, which can simply direct measurement without the need for calibration. Furthermore, an electrochemical sensor can also capture and transmit data digitally by wireless wearable electrochemical sensors that track the metabolic activity and physiological state with great resolution in remote areas [240]. In brief, electrochemical sensing relies on the electrochemical signal measurement technique in evaluating analyte perspiration; this sensor is suitable for applications needing large data for continuous measurement and measuring several analytes, with the electrode fabrication not exceeding three sensors. In addition, optical and electrochemical sensing can be combined to optimize the functioning of sensors. Kim et al. used hybrid colorimetric and electrochemical sensing integrated with a microfluidic device to provide the multimodal analysis of sweat glucose, lactate, chloride, pH, and sweat rate [172]. Remarkably, it realized visual and excitation light delivery with a detailed readable signal via the amperometry method.

Even when analyzing a single sweat application, integrating multiple sensors is needed to add important information, deeply understand the analyte reactions, and precisely analyze the disease conditions. For example, in kidney diagnosis, urea and creatinine sensors are used to diagnose kidney disease in sweat, while pH sensors can identify different stages of it chronically [58]. In another practice, the evaluation of calcium concentration requires pH measurement as an indicator of high and low calcium secretion in real time [62]. Sweat pH declines when the lactic acid concentration drops while the calcium concentration increases. On the other hand, the sweat pH is also relevant, coupled with the sodium concentration for monitoring hydration. pH measurement gives individual variability in the reproducible evaluation of sweat sodium concentration [241]. Occasionally, a sodium sensor is customarily hybrid with a potassium sensor [32], this being vastly exploited for wearable SSDs in sports analytics, while another study reported the mounting of sodium and ammonium sensors [33]. The association of multiform electrolyte sensors in a single SSD provides a better fitness performance analysis, which contributes to accurate measurements.

Additionally, an SSD that integrates with various chemical sensors can also be featured in multitasking sweat applications such as medical diagnostics and the analysis of fitness performance, to reduce time consumption by simultaneously analyzing the different purposes of sweat measurement. For instance, Choil et al. developed a sweat multiplexed sensing system that monitors pH, temperature, lactate, glucose, and chloride concentrations to detect changes in the concentration of these biomarkers corresponding with specific illness conditions and hydration levels [171]. Furthermore, Nyein et al. developed multiplex electrochemical sensors comprising pH, chloride ion, and levodopa composition sensing, which were used to associate with sweat released due to the response of physical and mental stress while the subjects were in a resting position [242]. When at rest, sweating more or less may be an additional sign of autonomic dysfunction, diabetes, cerebrovascular disease, Parkinson’s disease, and chronic psychological stress such as anxiety or pain [243,244]. Sweat pH can indicate acid–base imbalances [98], whereas chloride levels are helpful in testing for cystic fibrosis, electrolyte balance, and hydration status [214]. Sweat-based levodopa screening could contribute to precision therapy for Parkinson’s patients [4]. They optimally maximized multifunction sweat applications, including physical hydration, neurological afflictions, and mental condition.

In sweat analysis, adding physical/physiological signal sensors (e.g., blood pressure, heart rate, and electrocardiograms) that integrate with a chemical sensor can provide a sufficient overview of a patient’s health condition, the body’s response, and physiological changes to daily activities. Sempionatto et al. constructed an SSD that could monitor vital signs (blood pressure and heart rate), the concentration of metabolites (glucose and lactate), and exogenous chemical levels (caffeine and alcohol) by analyzing sweat extracted through exercise, iontophoresis, and external stimuli (intake of food, caffeine, and alcohol) [245]. These biomarker sensors were proposed to have a correlation with the health self-monitoring of common daily activities such as eating, drinking, and exercising. Their SSD was optimized to provide good mechanical resilience and a dependable glucose detection in sweat without crosstalk between the different sensors. In addition, Imani et al. proposed an epidermal patch that could continually track sweat lactate levels and ECG signals concurrently to assess a wearer’s physical strength, exercise intensity, and tissue oxygenation [246]. It is possible to monitor the health and function of the heart using electrocardiograms, while sweat lactate sensor integration can be used to monitor an individual’s fitness performance and diagnose tissue oxygenation and pressure ischemia. This device offers a practical approach to researching and developing multimodal wearable sensors that incorporate physical, electrophysiological, and chemical sensors to further measure human physiology comprehensively.

Table 2 shows the latest developed SSDs with various sensor features and measurements that have previously been discussed, such as solid contact materials, sensor types (electrochemical or colorimetric), and the incorporation of a physical/physiological signal sensor. These features are essential for determining whether the newest SSDs can be used repeatedly over the long term and whether they have already maximized their advanced performance by including multiple sensors, biophysical sensing, and multiple analyte measurement. The life span of the developed sensors was kept stable, with minimal drift of measurement values in current or voltage. In addition, blood testing, together with sweat analysis, can provide a good comparison and evaluate whether both biofluid samples have a strong correlation in order to check the reliability of the created sensor device and the practicability of the sweat sampling analysis for certain biomarkers [14,104]. Sweat has remained comparatively unexplored in comparison to standard biofluids, such as blood, despite its great promise for noninvasive physiological monitoring.

### 4.3. Electronic Device

Miniaturized electronic SSDs in the form of a simple and fashionable daily-worn accessory, such as a smartwatch, can be the ideal choice for a sweat monitoring platform routine. Cao et al. introduced a smartwatch integrated with a paper-based microfluidic patch for sweat monitoring, providing a digital display of real-time detection results of potassium and sodium ions in sweat [40]. However, the SSD they demonstrated was insufficient in accomplishing the specifications for a commercial product. More endeavors and clinical studies are needed to promote smartwatch intelligence, including incorporating additional sensors such as body temperature, pulse rate, and other chemical sensors. Lin et al. developed an SSD consisting of a microfluidic valve, sensors, and a wireless flexible printed circuit board to communicate with consumer electronic devices (e.g., smartwatches and smartphones through Bluetooth) [188]. Their smartwatch application includes the following three main functions that are accessible via the main selection screen, which also displays the current time: history (which stores and displays a time series bar chart that represents the most recently recorded biomarker data), scheduled (which exhibits the configured biomarker recording schedule and the sensor selection activation command, which includes glucose and lactate), and on-demand (a scrollable view from which the sensor activation command can be transmitted on-demand by a user who selects the required sensor section) [87,253].

Electricity generation for SSDs can be developed from battery-free systems, which offers miniaturized, lightweight, and wireless electronic devices that are more versatile under daily use. Typically, this electric energy can be retrieved from sustainable, portable, and renewable resources such as sunlight [254], human motion (biomechanical) [255], and biofuel cells (BFC) [256] to power future wireless wearable electronics. However, solar cells must be integrated with the energy storage devices of rechargeable batteries such as lithium-ion batteries, zinc-ion batteries, and supercapacitors, which contain hazardous constituent materials and require external charging or frequent power source replacement [3,128,257]. Moreover, energy storage devices and photovoltaic cells are required to address the limitations imposed by sunlight availability and serve as an efficient and long-term source of power [258]. Rechargeable batteries are also susceptible to explosion, generate huge currents, and are short-circuited when present with sweat, posing safety concerns, which may cause skin burns. Despite solar energy, human motion and BFC are viable alternatives. Both are types of on-body energy sources that are generally available and still in their infancy. Therefore, several biomechanical and biochemical system analyses are discussed in this section to provide concepts and insight for future researchers to optimize viable, ecologically friendly energy sources for future wearable electronics and support active device operation, raising intense research interest.

Energy harvesting from human motion generates electricity, which distributes the current into sensors and electronic devices through current displacement based on mechanical triggers. This is known as a nanogenerator, which is sorted into piezoelectric nanogenerators (PENG) [248,259] and triboelectric nanogenerators (TENG) [260,261]. Mechanical energy is an independent operation and is able to control the production of external power sources. The piezoelectric material adopted in PENGs is installed between the top and bottom electrodes. The electrostatic potential is generated by piezoelectric material, inducing an electric current to flow between both the electrodes when subjected to an exerted vertical strain. The electrons’ backflow creates a reversed current upon release of the strain. However, TENGs give a larger current output and can involve a wider range of material selections than PENGs, making it an excellent candidate for biomechanical energy harvesting in low-frequency human motion. Moreover, TENGs are flexible and versatile in recuperating the kinetic energy from the mechanical motion of various working modes of electrodes via the coupling of inductive and triboelectric effects [262,263], generating a flow of voltage and current. Song et al. proposed a freestanding TENG (FTENG)-powered SSD that efficiently extracts power from body motion, powering multiplexed sweat biochemical sensors and wirelessly transmitting health status tracking during exercise via Bluetooth to communicate with the user [218]. In comparison to the prior efforts of TENGs that suffer from low power intensity [264,265], their fabricated FTENG exhibited great significance in terms of mechanical and electrical reliability and stability, in which an outstanding performance in terms of real-time energy usage and longevity for wearable electronics was achieved. They proposed an efficient dielectric modulation strategy to tackle this challenge by optimizing their charge trapping capacity and surface charge density. Furthermore, the storage capacitor in the FTENG releases its stored energy when fully charged, maintaining optimum power over ultra-long charging periods to perform the measurement of practicality for wearable applications [266]. Meanwhile, some novel designs to enhance the output performance of PENGs still deserve more attention.

BFCs also carry a function as a self-powered sweat detecting system. The available target biomarkers in sweat secretion serve as fuels to transform the catalytic reaction into electrical power. Glucose [256,267,268] and lactate [269,270,271] are sweat analytes that consume biomass for electricity generation, benefitting the biocatalytic redox enzyme reaction to generate protons and electrons. Then, these charges in sweat become the external loading for the connection between the anode and cathode, producing a current intensity that is proportional to the biofuels’ concentration until the BFC is completely saturated. Sweat-lactate-based wearable biofuel cells are an effective candidate for addressing power concerns, owing to the fact that high lactate levels can be induced with low fitness levels, promising the production of a high-power density [269]. Besides glucose and lactate, sweat ethanol has also been tested as a fuel source in the creation of wearable biofuel cells [272]. A BFC based on in situ ethanol detection usually yields real-time bioelectricity generation from alcohol drinkers’ perspiration. Sweat from individuals consuming lower doses had lower alcohol absorption and excretion rates [273], leading to lower bioenergy harvesting from sweat, an important guideline for controlling alcohol ingestion for heavy drinkers. Sadly, enzymatic fuel cells cannot generate electricity for an extended period of time because many oxidation–reduction enzymes degrade rapidly with low interactions, limiting their biocatalytic activity, stability, endurance, and energy capacity. Interestingly, Ryu et al. pioneered high robustness and long-lasting perspiration-based electricity production via a wearable paper-based microbial fuel cell (MFC) made by using a spore-forming skin bacterium, Bacillus Subtilis (BS) [274]. In an extreme condition that led to the limitation of sweat, the BS formed endospores. When sweat was introduced, the BS repeatedly germinated spores to prevent their denaturation or degradation. This circumstance enabled the electrogenic capability of the BS to generate a sufficiently high-power density for a small-scale battery. Moreover, sensors powered by bacteria could be used to monitor human skin health and possibly release antibiotics, making BS a more appropriate source of energy when wound-healing devices or microneedles with transdermal drug delivery systems are present.

Recent wearable electronic devices in SSDs have incorporated wireless communication and transmission data using radio-frequency power communication technologies such as wi-fi, Bluetooth, and radiofrequency identification (RFID), which expose their users to radiation risks. However, there are currently no solid data regarding the hazards of low-level radiofrequency radiation, as chronic low-intensity radiation exposure poses unknown risks because of the lack of statistically significant effects in many studies, aside from the heating effects of excessive exposure [275]. However, when operating multiple numbers of high-power wearable or portable devices at the same time, researchers should be aware of the cumulative consequences of low-intensity radiation exposure [276]. These technologies are important in enabling the Internet-of-Things (IoT) that allows innovative sweat-sensing devices and other technology to communicate with one another over the internet. Nonetheless, everything involved with internet activity has the potential to erode user personal privacy including account details, passwords, and other vague data. Thus, user privacy must be protected using encryption schemes and personal identifiers for security purposes, whilst also maintaining safety from cyber attackers, scammers, and digital stalking. Abdulla et al. presented an IoT application in a sweating finger sensor that detected the user’s emotional state by utilizing a secure two-tier platform to integrate RFID and steganography to securely store collected data in a database while also increasing real-time data collection [277]. Steganography is a technique that encrypts user data to make it more secure than ever [278]. Consumers’ personal health assessments were stored in a separate database that could only be accessed by doctors and their families using RFID technology. RFID technology was used to ensure that the user can access the data only after placing their card on the reader, keeping the data in the IoT and a database.

Wearable sweat-sensing electronics have recently been established with machine learning (ML) to create smart and intelligent digital networks with wide-ranging applications in fields including healthcare monitoring, disease detection, and personalized medical treatment. An ML algorithm offers advanced tools for processing and analyzing a large volume and complexity of raw data from SSDs to improve the performance and quality of their system-level operation. This can be accomplished by combining it with statistical approaches that enable a computer to learn from past experiences/historical data, followed by the implementation of training and testing. The performance of the prediction model is measured using metrics such as mean absolute error (MAE) and standard deviation (SD). Baik et al. evaluated the performance of their colorimetric sensor by applying Pearson’s correlation between actual and predicted pH values [279]. The results of this analysis were r = 0.93; *p* < 0.01; MAE = 0.27. These data showed that their machine-learning-based pH quantification system was highly effective for accurately screening the skin’s pH and diagnosing the early signs of skin problems such as acne. They used linear regression to predict pH values from RGB value conversions. When the regression trend was linearly increasing, the color values were a significant indicator of pH. Their machine-learning-based software application could then automatically measure real-time pH values in situ based on the color displayed by the colorimetric pH monitoring of sweat. Using appropriate ML algorithms, important information about various signal characteristics can be extracted from raw data and exploited to their maximum capabilities. This would increase the intelligence of these wearable devices’ functionality. Lin et al. implemented a high recognition of ML on all-fiber motion sensors (AFMS) for relatively effective and precise tracking of human motion (e.g., throat) [280]. XGBoost (XGB), k-nearest neighbor (KNN), and support vector machine (SVM) are the three machine learning models that were utilized to classify the physiological data obtained from the AFMS for the purpose of detecting throat motion. XGB’s classification results were good, with few outliers, a concentrated distribution, a high mean value, and a high classification speed and accuracy. Thus, it was practicable and reliable for detecting the earliest signs of viral throat diseases, monitoring their severity, and diagnosing related symptoms. Other ML techniques for the most recent SSDs with some electronic device features are outlined in Table 3.

## 5. Conclusions and Future Research Directions

Human sweat biomarkers are significant indicators of a person’s physical state because they contain a wealth of physiological information that can be accessed non-invasively, eliminating the need for intrusive blood testing analysis. Various sweat biomarkers including electrolytes, metabolites, micronutrients, hormones, and exogenous chemical substances, have consistently drawn the attention of academic researchers for a variety of purposes and applications. Many studies have shown a strong high correlation between these sweat biomarkers concentration and those in blood, resulting in sweat measurement being reliable. In addition, the exploration of sweat analysis has brought forward the creation of SSDs that have demonstrated remarkable performance in the non-invasive, real-time detection of relevant analytes in sweat and have provided an opportunity for individuals to realize the dynamic tracking of their health status. SSDs are typically integrated with different types of wearable devices, sweat collection devices, sensors, and electronic devices. In this review, SSD categories, including CF and NCF, were introduced based on the concerns of mixing fresh and old sweat and real-time measurement, which are the requirements that ensure accurate measurement. Both categories feature a variety of sweat collection devices such as microfluidic devices with active/passive micropumps, absorption-based materials, and stimulant-based materials. In recent studies, various advanced features and technologies have been proposed to optimize the main components of developed SSDs.

Future research in sweat collection methods must address the low efficiency of sweat sampling collection, and the difficulty in accessing sweat production at rest while taking the consideration of patients, elders, and newborns. The incorporation of a chemical sweat-stimulation technique can provide solutions to these issues. A complex structure of microchannels is also suitable for handling very low sweat sample volumes. Additionally, researchers’ efforts in further works are required to investigate and analyze the specific differences in particular sweat compositions that are obtained from various sweat production processes. The traditional technologies of fabrication techniques for microfluidic devices, such as spin-coating and screen-printing, involve inefficient processing due to the excessive waste of raw materials. The advancement of 3D printing may provide a solution to this problem. Furthermore, 3D printing is better for processing the complex 3D structures of microfluidic devices and offers precise control over the shape and spatial dimensions of a microchannel. Moreover, novel materials and efficient fabrication techniques need to be broadened in terms of exploration to achieve the requirements of advanced wearable sensors, such as ultra-sensitivity, robustness (i.e., maintaining stability with good sensitivity after repeated and long-term use), the incorporation of vital signs to provide more detailed information of personalized healthcare, high selectivity in multiple analyte measurement, and mass production. In addition, integrating artificial intelligence with SSDs is promising for accurate data processing and healthcare decision-making. Moreover, it can provide dependable, secure, and accessible data to medical experts so that they can make clinical decisions precisely. Lastly, for any wearable electronic device, an equipment-free and environmentally friendly power supply, such as BFCs and TENGs, must be developed as its source must is always available. This will enhance automatic self-powered sensors while addressing the concerns surrounding required repeat charging, bulky size, and safety issue. Overall, this review critically discussed the analytical requirements for reliable guiding and benefits future development toward precision health monitoring.

## Figures and Tables

**Table 1:** A summary of the latest SSDs’ sweat collection device features advancements in 2022.

Categories of Sweat Collection Devices	SSD	Wearable Devices	Dimensions/Depth of Channel	Flow Rate and Time to Fill Channel	Reusable/Disposable	Additional Features	Validation Method	Mechanical Testing	References
Microfluidic device, portable iontophoresis of pilocarpine, adding hydrogel	CF	Epidermal patch	5 mm (Outer diameter), 1 mm (Inner diameter)	N/A 15 min (Total volume 32 µL)	Reusability	Multiple inlets (n = 3)	On-body test	Bending, stretching, twisting	[14]
Modification of hydrophobic microfluidic device to a hydrophilic surface	CF	Epidermal patch	10 mm (diameter), 1 mm (thickness)	0.05–0.5 m/s (Total volume 200μL)	Reusability	Tesla valves	Simulation, off-body test, on-body test	N/A	[136]
Microfluidic device	CF	Epidermal patch	N/A	3–12 mm/s 13 min for indoor exercise, 20 min for outdoor exercise	Reusability	Multiple inlets (n = 3)	Off-body test, on-body test	Bending, stretching, twisting, tensile	[200]
Modification of hydrophobic microfluidic device to a hydrophilic surface	CF	Epidermal patch	1.5 mm (Inlet diameter), 4 mm (Reservoir diameter), 200 µm (thickness)	0.14 μL/min (each inlet), 0.84 μL/min (total) 14 min (Total volume 11.8 µL)	N/A	Multiple inlets (n = 6)	Simulation, off-body test, on-body test	Bending, pressing	[201]
Microfluidic device	CF	Epidermal patch	1 mm (diameter), 330 μm (thickness)	174.6 μL/min (Total volume 20μL)	N/A	Capillary bursting valves, colorimetric, multiple inlets (n = 8)	Simulation, off-body test, on-body test	Bending, stretching, twisting	[202]
Microfluidic device, absorptive pad	CF	Epidermal patch	N/A	5 μL/min (Total volume 10μL)	Reusability	Suction pump reset after filling the channel	Off-body test, on-body test	N/A	[203]
Textile	NCF	Stitched fabric with three button joints	N/A	N/A	Reusability	Washability	On-body test	Washing, drying (thermal)	[204]

**Table 2:** A summary of the latest SSDs’ sensor features advancements in 2022.

Analytes Detection	Solid Contact Materials	Types of Sensors	Physical/ Physiological Signal Sensor	Sample	Correlate with Blood Test	Techniques Measurements	Repeatability and Life Span	References
Levodopa	Zeolitic imidazolate framework/ graphene oxide (ZIF-8/GO)	Electrochemical	N/A	Sweat	Yes	Chronoamperometry, cyclic voltammetry	N/A 7 days	[247]
Glucose	PB	Colorimetry, electrochemical	N/A	Sweat, blood	Yes	Amperometry	N/A	[14]
Lactate	N/A	Colorimetric	Temperature	Artificial sweat, human sweat	Yes	Convolutional neural networks (CNNs)	N/A	[94]
Glucose, pH	PANI, reduced glucose oxidase (GOx)/ PtNPs/ Gold (Au)	Electrochemical	ECG, temperature, heart rate	Artificial sweat, human sweat, blood	Yes	Amperometry, potentiometry	Repeatability 1 week	[104]
$Cl^{-}$*,* pH	N/A	Colorimetric	N/A	Artificial sweat, human sweat	N/A	Color intensity changing (absorbance, wavelength)	N/A	[202]
Glucose	PB-PEDOT-N	Electrochemical	N/A	Sweat, blood	Yes	Chronoamperometry	Repeatability 1 month	[209]
$Na^{+}$ $,\;K^{+}$	PEDOT/PSS	Electrochemical	N/A	Sweat	N/A	Chronoamperometry, potentiometry	Repeatability -	[215]
$Na^{+}$$,\;K^{+}$*,* pH	PEDOT/PSS, PANI	Electrochemical	N/A	Sweat	N/A	Chronoamperometry, potentiometry	Repeatability 30 days	[248]
$Na^{+},\;K^{+},\;Pb^{+2},Li^{+}$	Platinum nanoparticles (PtNPs)	Electrochemical	Temperature	$\mathrm{NaCl},\;\mathrm{KCl},\;\mathrm{LiCl},\;\mathrm{Pb}(NO_{3}{)}_{2}$	Yes	Potentiometry	N/A	[249]
Glucose, lactate	PB	Electrochemical	Heart rate	Sweat	Yes	Amperometry	Disposable	[250]
Uric acid	Metal azolate framework-7 (MAF-7)	Electrochemical	N/A	Artificial sweat, human sweat	N/A	Amperometry, cyclic voltammetry	N/A	[251]
Creatinine	N/A	Fluorescence	N/A	Sweat, urine	Yes	Color intensity changing (absorbance, wavelength)	N/A	[252]

**Table 3:** A summary of the latest SSDs’ electronic device features advancements in 2022.

SSD Forms	Types of Power Source	Sensor Involved	Wireless Communication	Commercial Product	Machine Learning	Applications	References
Smartwatch	Rechargeable LiPo battery	Temperature sensor, relative humidity sensor, glucose sensor	Bluetooth	N/A	Decision tree regression algorithm	Continuous glucose monitoring	[281]
Adhesive tape	TENG	Acoustic sensors, epidermal sensor, triboelectric sensor, heart rate sensor	Internet-of-Things (IoT)	N/A	Deep learning algorithms	Human activity monitoring, cardiovascular monitoring, acoustic-biometric applications	[282]
Smart necklace	BFC	Sodium, hydrogen, potassium, glucose sensor	Vector network analyzer (VNA)	N/A	A low-pass fast Fourier transform algorithm	Detect sweat electrolytes and glucose	[283]
Hexagonal bounding shape of microfluidic patch	N/A	Colorimetric, sodium sensor, chloride sensors	Image capture from microfluidic patch sweat metrics using smartphone	N/A	Canny edge detection algorithm, image analysis algorithms, multiple regressions	Sweating rate, total sweat loss, sweat electrolyte concentration loss	[284]
A nano-porous polyamide substrate along with serpentine gold electrodes	Battery	Cytokine sensor	N/A	N/A	Supervised discriminant factor analysis (DFA) linear regression model of a binary classifier	Detect of Interleukin-31 (IL-31), chronic skin disease	[285]
Wristband	3.7 V LiPo battery (168 h on single charge)	Interferon-inducible protein (IP-10), tumor necrosis factor- related apoptosis-inducing ligand (TRAIL), and C-reactive protein (CRP) sensors	Bluetooth (Smartphone app)	SWEATSENSER Dx-EnLiSense	N/A	Detect simultaneously and continuously specific IP-10, TRAIL, CRP	[286]
Smartwatch	110 mAh Li-ion battery	Cortisol sensor	Bluetooth	Aptamer-FET biosensing smartwatch	N/A	Track stress level	[287]
Wristband	N/A	IL-6 sensor, pH sensor	Bluetooth	WRRIST	N/A	Detect IL-6 levels (Inflammatory biomarkers)	[288]

## Data Availability

Not applicable.
